# Consensus on Severity for Ocular Emergency: The BAsic SEverity Score for Common OculaR Emergencies [BaSe SCOrE]

**DOI:** 10.1155/2015/576983

**Published:** 2015-07-30

**Authors:** Jean-Louis Bourges, Isabelle Boutron, Dominique Monnet, Antoine P. Brézin

**Affiliations:** ^1^Department of Ophthalmology, Paris Descartes School of Medicine, Assistance Publique-Hopitaux de Paris, Cochin-Hôtel-Dieu Hospital, Université Sorbonne Paris Cité, 75004 Paris, France; ^2^INSERM U1138 Team 17, Le Centre de Recherches des Cordeliers (CRC), 75006 Paris, France; ^3^Department of Biostatistics, Paris Descartes School of Medicine, Assistance Publique-Hopitaux de Paris, Cochin-Hôtel-Dieu Hospital, Université Sorbonne Paris Cité, 75004 Paris, France

## Abstract

*Purpose*. To weigh ocular emergency events according to their severity.* Methods*. A group of ophthalmologists and researchers rated the severity of 86 common ocular emergencies using a Delphi consensus method. The ratings were attributed on a 7-point scale throughout a first-round survey. Then, the experts were provided with the median and quartiles of the ratings of each item to reevaluate the severity levels being aware of the group's first-round responses. The final severity rating for each item corresponded to the median rating provided by the last Delphi round.* Results*. We invited 398 experts, and 80 (20%) of them, from 18 different countries, agreed to participate. A consensus was reached in the second round, completed by 24 experts (43%). The severity ranged from subconjunctival hemorrhages (median = 1, Q1 = 0; Q3 = 1) to penetrating eye injuries collapsing the eyeball with intraocular foreign body or panophthalmitis with infection following surgery (median = 5, Q1 = 5; Q3 = 6). The ratings did not differ according to the practice of the experts.* Conclusion*. These ratings could be used to assess the severity of ocular emergency events, to serve in composite algorithms for emergency triage and standardizing research in ocular emergencies.

## 1. Introduction

The modern management of ocular emergencies requires growing fields of expertise to implement appropriate triage of patients [1–6]. A correlation between final visual outcome and prompt access to specialized care has been reported [7–10]. One of the most popular tools in Emergency Eye Clinics (EEC) relies on the first level of decision made by an Ophthalmic Nurse Practitioner (ONP) [5, 11, 12]. The patient referred to EEC may eventually be oriented to an ophthalmologist within an arbitrary defined time frame. The New South Wales (NSW) Health and State and the NSW Statewide Ophthalmology Service of Sydney have released recommendations to manage ocular emergency, aiming at implementing some consensual procedures and care [13]. The first formal eye-dedicated triaging system, the RESCUE system, was proposed in 2007 by Rossi et al. [4]. It consists of a color-coding scheme discriminating cases through three grades of severity and promptly identifies those who eventually might require hospitalization. However, patients are also commonly referred to EEC for ocular emergencies by a first-line health care practitioner for further specialized eye care and appropriate treatment. At that time point, an identification of the ocular condition is usually tentatively proposed or at least suspected. Thereafter, the triage efficiency would dramatically benefit from a basic but consensual tool scoring severity for each possible ocular emergency item presenting at emergency room. In addition, such a tool would help manage the downstream of eye emergency. Finally, a consensus tool scoring severity of eye emergency items would eventually serve for research purposes to weigh the severity of rather heterogeneous items in clinical trials.

We were therefore driven to reach a consensus based on the severity of the most common ocular conditions seen at EEC, aiming at attributing to each of them a simple clinical score for severity.

## 2. Methods

We built the BAsic SEverity Score for Common OculaR Emergencies [BaSe SCOrE] on the basis of a comprehensive survey conducted with a panel of experts in the field of ophthalmology and/or emergency eye care.

### 2.1. Study Design

The Delphi method was used to synthesize expert opinions and achieve consensus [14, 15]. Briefly, it entailed a panel of experts who answered the BaSe SCOrE survey and subsequently received feedback on the “group response,” after which the process was repeated again to reach a majority agreement. The Delphi consensus relies on an iterative, anonymous method with controlled feedback and statistical aggregation of group responses [16, 17].

### 2.2. Steering Committee

A steering committee including three ophthalmologists (M.D., Ph.D.) (Jean-Louis Bourges, Dominique Monnet, and Antoine P. Brézin), one epidemiologist (M.D., Ph.D.) (Isabelle Boutron), and two nurses (Catherine Issad and Michel Alvarez) participating in the ocular emergency health care system was built.

### 2.3. Emergency Item Identification

Ocular emergency items were listed on the basis of the relevant literature to be further proposed in the survey. The first author reviewed the literature published before August 2012 and available on PubMed, Cochrane Library, and Google Scholar websites. The URLs http://www.ncbi.nlm.nih.gov/entrez/query.fcgi?DB=pubmed, http://www.thecochranelibrary.com/view/0/index.html, and http://scholar.google.fr/schhp?hl=fr were visited using the following keywords: eye and (emergenc^*∗*^ or trauma^*∗*^)/ocular^*∗*^ and (emergenc^*∗*^ or trauma^*∗*^) and (French [Language] or English [Language]). The star was used as a wildcard. The relevant literature was selected on the basis of paper titles and abstracts. Every generic term pointing to an ocular condition related to emergency care was considered a possible item, captured and sorted into four main categories: anterior segment items, posterior segment items, traumatic items, and items linked to complications following ocular surgery. The list of items was completed, amended, and eventually validated by the steering committee. Finally, the steering committee approved a list of 86 items, classified as 21 anterior segment items, 26 posterior segment items, 25 traumatic items, and 14 items related to complications after ocular surgery. The list was further proposed in the survey. In the survey, the formulation of items was open to free comments from participating experts to ensure the relevance and reliability of the final scoring and to strengthen the consensus.

### 2.4. Selection of the Panel of Experts

We systematically selected all the email addresses of the corresponding authors of the original articles available in full text and obtained as described above. Further, we systematically selected all corresponding authors with an email address available from an original article published between 2011 and 2012 in six scientific journals (Ophthalmology, Investigative Ophthalmology and Visual Science, Archives of Ophthalmology, British Journal of Ophthalmology, American Journal of Ophthalmology, and Journal of Cataract and Refractive Surgery), rated within the top ten journals for their impact on the field of ophthalmology. We included Ph.D., O.D., or nonclinician health care practitioners in the panel of experts if they were involved in the field of ocular emergency, referring patients to EEC or participating in dedicated research.

### 2.5. Delphi Consensus Method

We planned to conduct at least two iterative rounds by email, using the SurveyMonkey tool. Additional rounds could be performed until a consensus was reached. Email addresses for the selected experts were extracted from their publications, and they were invited to participate online in the Delphi process. They were sent a standardized information package, including a synopsis of the study and a description of the Delphi process. Experts who did not respond were solicited again by an email reminder sent four days, two weeks, and four weeks after the initial invitation, before being considered permanently unwilling to participate. Experts who declined our invitation were asked to recommend the best expert in the field of ocular emergency. Recommended persons were invited following an identical procedure. The purpose and the methodology of the survey were further detailed for the experts who agreed to participate by providing remote access to a dedicated website. The opportunity to decline to participate permanently was offered to the experts.

In our first Delphi round, each member of the panel evaluated the severity of the 86 items based on a 7-point scale (Supplementary Material available online at http://dx.doi.org/10.1155/2015/576983). For each event, experts were asked the following question: “According to your personal expertise, please rate the severity of the following ocular conditions seen in the emergency room, using the following seven-point ordinal scale.” The 7-point scale was used to collect responses with the anchors “not severe at all” at 0 and “maximal (currently untreatable)” at 6. Severity was left to each expert's own understanding. Within the survey, a dedicated email address and some free writing space were offered to harvest the experts' comments and requests. Thus, the experts could comment or suggest items freely and possibly modify items in the next round with the approval of the steering committee. At the end of the first round, the steering committee elaborated a synthesis of the collected data.

In the second round, the experts were informed of each median rating (Q1–Q3) collected from the first round. Considering the same event, the same question, and the same rating scale, the experts were asked to rate each event again by confirming their first rating or modifying it in the light of the first-round results.

### 2.6. Data Analysis

The answers were collected, reviewed, and analyzed by the steering committee (Jean-Louis Bourges, Isabelle Boutron, Dominique Monnet, Antoine P. Brézin, Catherine Issad, and Michel Alvarez). We applied a last observation carried forward (LOCF) strategy for missing data after the first round. Thus, if an expert did not answer the second round, we considered his/her first-round answer. The concept of consensus within a group was defined as homogeneity or consistency opinion among the experts. Assuming that each item was characterized by a constant but unknown severity, the ratings of the experts could be considered multiple measures of this characteristic. The median rating and the first quartile–third quartile [Q1–Q3]) were established considering each individual item for the whole group.

The final weight for each event was the median rating obtained during the last Delphi round. All analyses were performed on the R statistics system (version 2.10.0; https://www.r-project.org/).

## 3. Results

### 3.1. Participants

**Figure 1:**
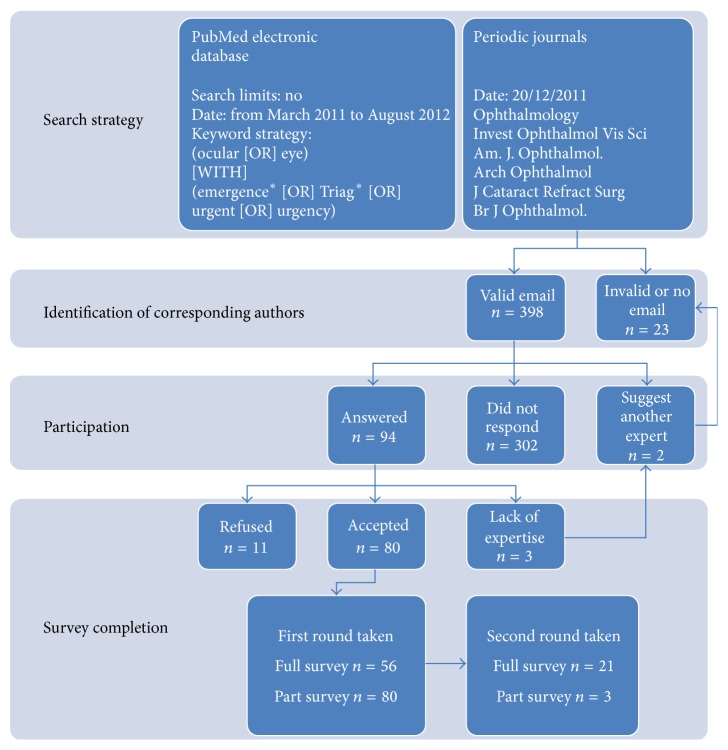
Flow chart of the Delphi process for the BaSe SCOrE.

We invited 398 experts to participate (Figure 1). A fifth took the first round of the survey (*n* = 80), answering from 18 different countries.  Table 1 displays the preferred practice of the participating experts. The experts completing the first round claimed an M.D. (67%), M.D. and Ph.D. (25%), or Ph.D. (4%) professional degree. The other reported professional degrees were optometrist (O.D.) and various other scientific degrees. Fifty-six experts scored all 86 items in the first round and all experts scored all items in round 2. Twenty-four experts (43%) completed the second round.

### 3.2. BaSe SCOrE Consensus

**Figure 2:**
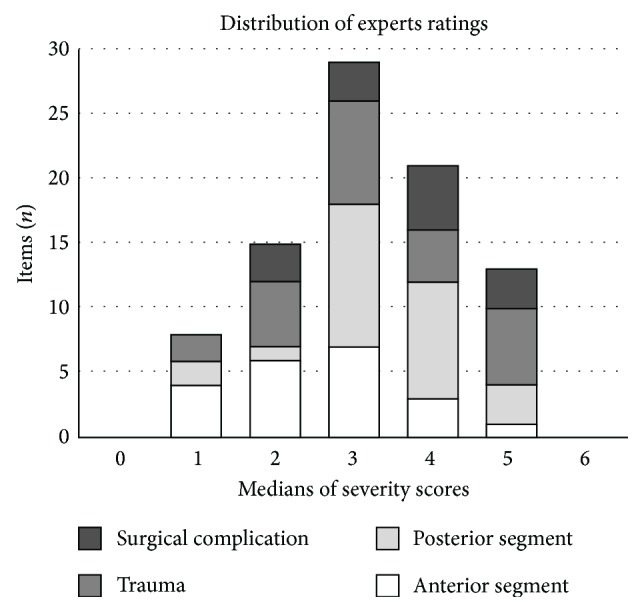
Distribution of the severity score medians weighted from 0 to 6, obtained by consensus among the four proposed categories of items (anterior segment, posterior segment, traumatisms, and complication of ocular surgical procedure).

Table 2 reports the consensus reached by the experts who ranked the global severity of each item within the four proposed categories. For anterior segment items, the experts considered noninfectious conjunctivitis as the less severe event (median = 1 [Q1 = 1; Q3 = 1]), whereas perforating ulcer of the cornea was ranked as the most concerning item (median = 5 [Q1 = 5; Q3 = 5]). Subconjunctival hemorrhage, classified as a posttraumatic event, was considered the least severe item (median = 1 [Q1 = 0; Q3 = 1]). For items involving the posterior segment, ophthalmic migraine and vitreous floaters were both ranked as the least severe items (median = 1 [Q1 = 1; Q3 = 2]), although they were considered slightly more severe than noninfectious conjunctivitis. Retinal artery occlusion was considered the most severe item (median = 5 [Q1 = 4; Q3 = 6]), ahead of retinal detachment and acute binocular diplopia with neurological symptoms (median = 5 [Q1 = 4; Q3 = 5]). Among traumatic items, penetrating eye injuries either with intraocular foreign body (median = 5 [Q1 = 5; Q3 = 6]) or without intraocular foreign body (median = 5 [Q1 = 5; Q3 = 5]) were ranked highest like panophthalmitis from items following intraocular surgery. The second round only modified 3 median scores with quartiles remaining unaltered: subconjunctival hemorrhage (0.0 to 1.0), retinal detachment (macula off) (5.0 to 4.0), and ophthalmic migraine (2.0 to 1.0).  Figure 2 illustrates how the severity ranking was distributed among the four main categories of items. No 0 or 6 median scores were obtained. The median score attribution seemed to follow a Gaussian-shaped distribution.

## 4. Discussion

The BAsic SEverity Score for Common OculaR Emergencies [BaSe SCOrE] is, to our knowledge, the first attempt obtained by expert consensus to rate ocular emergency items for severity. No unexpected or surprising rating emerged from the BaSe SCOrE. Actually, consensual scores obtained from the Delphi method faithfully transpose the reality of a day-to-day ophthalmological practice which includes eye emergencies. It seems obvious to every ophthalmologist that, regarding severity, conjunctivitis should be less rated than any acute anterior uveitis. Yet, there had been so far no tool to approach the difference between these two items quantitatively. The present score offers the opportunity to ophthalmological staff members, health care practitioners, or researchers to quantify ocular severity of frequent emergency conditions reproducibly, reliably, and most probably accurately. We foresee the core utility of the BaSe SCOrE both for emergency room staff to triage patients who are suspected to have a specific ocular condition and to standardize research in ocular emergencies. The way this score could be integrated in the triage process still remains to be defined. Even if it is essential, the BaSe SCOrE stays a preliminary step.

It may be also extremely difficult to discriminate severity among miscellaneous items, such as branch retinal vein occlusion (BRVO) and perforating but self-sealing laceration of the cornea. Not only are such items medically highly unrelated, but the actual severity of ocular conditions could at best be approached at the subspecialty level. According to the BaSe SCOrE consensus, the BRVO and the corneal perforation would both fall into the same quartile range, but the former would be given a median score of 3, while the latter would score 4. Thus, the global approach of the consensus demonstrates the ability to weigh the severity of items in situations where physicians would encounter difficulties assessing it.

Throughout the survey, some clinical items were purposely vaguely or rather imprecisely described. The deliberate choice to define each item in one way or another was made to best approach each situation encountered in emergency room.

Similarly, we chose not to define the word “severity” precisely during the consensus process. Severity is a broad term that encompasses several concepts, including the decisive role of immediate care, every foreseeable outcome, invasiveness of treatments, room for worsening, contagiousness, pain, or associated injuries. By taking the survey, each expert had the opportunity to consider every relevant notion, selecting his or her own score. By scoring ocular emergency items in relation to each other and by using severity as a term with a wide meaning, we applied the heuristic method. We believe it generates optimal results for clinical classification, rendering it more relevant in real-life practice and more realistic to implement the BaSe SCOrE at EEC.

Managing an ocular emergency, just like managing other emergencies, usually requires following three main steps: immediate triage, patient examination, and downstream orientation. The first-line triage mainly relies on patient's medical history and presenting symptoms. The anamnesis is collected widely by nonmedical staff, who optionally refer patients to the specialist, usually using a formalized protocol. In 2006 in Florida, less than half of the ophthalmologists answered emergency department calls [18]. Consequently, patients are oriented within a preset deadline by various health practitioners, who may have a medical degree or simply belong to the paramedic staff with various levels of experience [19]. We postulate that the different health care providers would interact in a rationalized, secured, and even more coherent fashion by using a standard score for severity. Communication is now strongly increased by the use of smartphones, tablets, and laptops through medical applications [20]. Using mobile devices, the BaSe SCOrE could improve the efficiency of triage by giving health care practitioners a consensual tool to standardize their decision making process.

The eye-dedicated RESCUE triage [4] used color coding to identify the acceptable delay for patients to have access to eye care. The triaging system does not integrate a quantified tool to evaluate the severity of ocular items. To be implemented reproducibly worldwide, this system as well as other ocular emergency triage systems could be fine-tuned by integrating the quantitative rating for severity of common emergency items generated by the BaSe SCOrE.

The present consensus displays strengths and limitations. The large number of experts willing to participate in the process makes the mean scores fairly reliable. The extended range of the experts' affiliations and participating countries adds to the robustness of the BaSe SCOrE, which might fit every EEC worldwide, independently of inherent specificity of local health care systems. The consensus form gave experts the opportunity to provide open comments. However, only two comments were gathered throughout the survey process. This could indicate that the BaSe SCOrE items and groups of items were accurate and meaningful to a wide range of eye care professionals from various countries. On the other hand, participation in the second round was limited. This could have resulted from the experts' agreement to the first-round results, provided at the time of the second survey. At that point, the experts might have felt no need to contribute further to the process by completing the second round, arguing that they would not change their minds on the basis of the knowledge of the first-round mean scores. It may also have resulted from experts' weariness in taking time from their busy schedules to complete surveys. Finally, the BaSe SCOrE still needs to be validated in EEC actual practice with a test sample of patients to assess its clinical accuracy by correlating the scores with the real clinical outcomes.

## Supplementary Material

The BaSe SCOrE survey consisted of an online form send to the experts. Participating experts completed the first round of the Delphi process provided in appendix 1. A second online form (appendix 2) was send to the expert based on the first form augmented with the median scores and quartiles resulting from the first round.

## Figures and Tables

**Table 1:** Overview of preferred practices and experience claimed by participating experts.

	Professional degree						
	M.D.	Ph.D.	M.D.-Ph.D.	O.D.	Other	*N*	%
Country of the professional institution							
USA	19	2	4		2	27	34%
France	9		4			13	16%
Australia	4		2			6	8%
Italy	4		1			5	6%
UK			4		1	5	6%
Netherlands	2		2			4	5%
India	3					3	4%
Taiwan	3					3	4%
Japan	1		2			3	4%
Greece	1		1			2	3%
Brazil		1	1			2	3%
Others	4		2	1		7	9%
Answers (*n*)	**50**	**3**	**23**	**1**	**3**	**80**	**100%**

Preferred practice							
Cornea and external disease	16		3			19	24%
Vitreoretinal diseases	12		7			19	24%
Glaucoma	6	2	3			11	14%
Cataract and refractive surgery	4		5			9	11%
Pediatric ophthalmology	5		1			6	8%
Ophthalmic plastic surgery	3		1		1	5	6%
Other	3	1	3	1	2	10	13%
Answers (*n*)	**49**	**3**	**23**	**1**	**3**	**79**	**99%**

Professional experience							
1 to 5 years	14	1	8		1	24	30%
11 to 20 years	13	1	6	1		21	26%
6 to 10 years	8	1	3			12	15%
Less than a year	3		1			4	5%
More than 20 years	11		5			16	20%
Not answered						3	4%
Answers (*n*)	**49**	**3**	**23**	**1**	**1**	**80**	**100%**

**Table 2:** Consensus reached on items from the 4 main categories of ocular emergencies and sorted by median weight rating severity.

Category	Item	Opinion submitted		Median weights	Quartile [1st; 3rd]
		Yes (*n*)	No (*n*)		
Anterior segment items					
Conjunctivitis	Noninfectious	74	6	1	[1.0; 1.0]
Keratitis	Superficial punctate keratitis	73	7	1	[1.0; 1.0]
Contact lens	Mechanical complication (dislocation, vacuum, etc.)	74	6	1	[1.0; 2.0]
Pterygia	Inflamed pterygia	74	6	1	[1.0; 2.0]
Conjunctivitis	Viral conjunctivitis	74	6	2	[1.0; 2.0]
Scleral and episcleral inflammation	Episcleritis (regardless of the cause)	74	6	2	[1.0; 2.0]
Corneal ulcer (not infected)	Unperforated—isolated epithelial defect	73	7	2	[1.0; 3.0]
Conjunctivitis	Bacterial conjunctivitis	74	6	2	[2.0; 2.0]
Keratitis	Noninfectious interstitial keratitis	72	8	2	[2.0; 3.0]
Anterior acute uveitis	First episode (regardless of the cause)	72	8	2	[2.0; 3.0]
Contact lens	Infectious keratitis without severity factor	74	6	3	[2.0; 3.0]
Anterior acute uveitis	Iterative (regardless of the cause)	73	7	3	[2.0; 3.0]
Scleral and episcleral inflammation	Scleritis (regardless of the cause)	74	6	3	[2.0; 3.0]
Lacrimal ducts	Acute dacryocystitis	73	7	3	[2.0; 3.0]
Corneal ulcer (not infected)	Unperforated, with stromal involvement	74	6	3	[2.0; 4.0]
Keratitis	Infectious keratitis	73	7	3	[3.0; 4.0]
Ocular surface burn	<9 clock hours of limbus and <75% of conjunctiva	73	7	3	[3.0; 4.0]
Contact lens	Infectious keratitis with severity factor(s)	74	6	4	[4.0; 5.0]
Glaucoma	Acute angle-closure glaucoma	73	7	4	[4.0; 5.0]
Glaucoma	Neovascular glaucoma	73	7	4	[4.0; 5.0]
Corneal ulcer (not infected)	Perforating ulcer	74	6	5	[5.0; 5.0]

Posterior segment items					
Nonspecific visual symptoms	Ophthalmic migraine	70	10	1	[1.0; 2.0]
Nonspecific visual symptoms	Vitreous floaters	70	10	1	[1.0; 2.0]
Choroidal new vessels (CNV) or direct complication	Peripheral (exclusively)	70	10	2	[2.0; 3.0]
Macula	Macular edema	71	9	3	[2.0; 3.0]
Chorioretinal toxoplasmosis (active phase)	Peripheral	71	9	3	[2.0; 3.0]
Pupil disorders	No oculomotor disturbance	71	9	3	[2.0; 3.0]
Retinal peripheral tear	No detached edges, no RD associated	70	10	3	[2.0; 3.0]
Chorioretinal toxoplasmosis (active phase)	Vitreous hemorrhage—isolated/unidentified cause	71	9	3	[2.0; 4.0]
Acute binocular diplopia	Without neurological symptoms	71	9	3	[2.0; 4.0]
Macula	Macular hole	70	10	3	[2.0; 4.0]
Optic neuritis (regardless of the cause)	Without neurological symptoms	71	9	3	[3.0; 4.0]
Retinal vein occlusion	Branch (BRVO)	70	10	3	[3.0; 4.0]
Retinal peripheral tear	Detached edges, no RD associated	70	10	3	[3.0; 4.0]
Posterior segment inflammation	Vitritis (unidentified etiology)	70	10	3	[3.0; 4.0]
Posterior segment inflammation	Retinal vasculitis	71	9	4	[3.0; 4.0]
Retinal artery occlusion	Branch (BRAO)	70	10	4	[3.0; 4.0]
Choroidal new vessels (CNV) or direct complication	Subfoveal	70	10	4	[3.0; 4.0]
Optic neuritis (regardless of the cause)	With associated neurological symptoms	71	9	4	[3.0; 5.0]
Pupil disorders	Associated with oculomotor disturbance	71	9	4	[3.0; 5.0]
Retinal vein occlusion	Central (CRVO)	70	10	4	[3.0; 5.0]
Ischemic optic neuropathy	Isolated	69	11	4	[3.0; 5.0]
Retinal detachment (RD)	Macula off	71	9	4	[4.0; 5.0]
Chorioretinal toxoplasmosis (active phase)	Foveal or peripapillary	71	9	4	[4.0; 5.0]
Retinal detachment (RD)	Macula on	71	9	5	[4.0; 5.0]
Acute binocular diplopia	With associated neurological symptoms	71	9	5	[4.0; 5.0]
Retinal artery occlusion	Central (CRAO)	69	11	5	[4.0; 6.0]

Traumatisms items					
Conjunctiva	Subconjunctival hemorrhage	71	9	1	[0.0; 1.0]
Conjunctiva	Conjunctival wound without scleral exposure	71	9	1	[1.0; 2.0]
Corneal injury	Corneal foreign body away from the axis	71	9	2	[1.0; 2.0]
Other injuries	Eyelid skin injury	71	9	2	[1.0; 2.0]
Conjunctiva	Conjunctival wound with scleral exposure	71	9	2	[2.0; 3.0]
Corneal injury	Nonperforating laceration away from the axis	71	9	2	[2.0; 3.0]
Commotio retinae	Peripheral posttraumatic retinopathy	70	10	2	[2.0; 3.0]
Corneal injury	Corneal foreign body in the visual axis	71	9	3	[2.0; 3.0]
Blunt injuries with impaired vision	Hyphema (isolated)	71	9	3	[2.0; 3.0]
Other injuries	Eyelid-margin injury	71	9	3	[2.0; 3.0]
Other injuries	Eyelid levator injury	71	9	3	[3.0; 3.0]
Corneal injury	Nonperforating laceration in the visual axis	71	9	3	[3.0; 4.0]
Commotio retinae	Posttraumatic maculopathy	70	10	3	[3.0; 4.0]
Blunt injuries with impaired vision	Iridodialysis (avulsion of the iris root)	71	9	3	[3.0; 4.0]
Other injuries	Tear ducts injury	71	9	3	[3.0; 4.0]
Corneal injury	Perforating but self-sealing laceration	70	10	4	[3.0; 4.0]
Blunt injuries with impaired vision	Choroidal rupture	71	9	4	[3.0; 4.0]
Blunt injuries with impaired vision	Lens dislocation	71	9	4	[3.0; 5.0]
Penetrating eye injury	Without intraocular foreign body, eye not collapsed	71	9	4	[4.0; 5.0]
Corneal injury	Perforating and leaking laceration	71	9	5	[4.0; 5.0]
Penetrating eye injury	With intraocular foreign body, eye not collapsed	71	9	5	[4.0; 5.0]
Blunt injuries with impaired vision	Scleral rupture	71	9	5	[4.0; 5.0]
Other injuries	Optic nerve injury	71	9	5	[4.0; 5.0]
Penetrating eye injury	Without intraocular foreign body, eye collapsed	71	9	5	[5.0; 5.0]
Penetrating eye injury	With intraocular foreign body, eye collapsed	71	9	5	[5.0; 6.0]

Items from complication following ocular surgery					
Acute pain	With normal postoperative course	69	11	2	[2.0; 3.0]
High intraocular pressure (except AACG)	25 < IOP < 35 mmHg	69	11	2	[2.0; 3.0]
Suture-related complication	Noninfectious suture-related complication	70	10	2	[2.0; 3.0]
High intraocular pressure (except AACG)	IOP = 35 mmHg or IOP > 35 mmHg	67	13	3	[3.0; 4.0]
Laser keratomileusis in situ (LASIK)	Complication related to the flap	70	10	3	[3.0; 4.0]
Keratoplasty (corneal grafting procedure)	Suture-related complication (no infection)	70	10	3	[3.0; 4.0]
Intraocular hemorrhage	Within 3 days after the surgery	70	10	4	[3.0; 4.0]
Keratoplasty (corneal grafting procedure)	Acute rejection	70	10	4	[3.0; 5.0]
Mechanical complication following surgery	Reopening of the surgical wound	68	12	4	[3.0; 5.0]
Suture-related complication	Infectious suture-related complication	69	11	4	[3.0; 5.0]
Mechanical complication following surgery	Collapsed eyeball (athalamia)	61	19	4	[4.0; 5.0]
Keratoplasty (corneal grafting procedure)	Graft dislocation	70	10	5	[4.0; 5.0]
Infection following surgery	Blebitis	70	10	5	[4.0; 5.0]
Infection following surgery	Panophthalmitis	70	10	5	[5.0; 6.0]
