# Supplementary material for: Consensus on Severity for Ocular Emergency: The BAsic SEverity Score for Common OculaR Emergencies [BaSe SCOrE]
Source: J Ophthalmol. 2015 Jul 30;2015:576983. doi: 10.1155/2015/576983 (PMC4534620; doi:10.1155/2015/576983)

Consensus on scoring severity of ocular emergencies: the BAsic SEverity Score for Common OculaR Emergencies [BaSe SCOrE]

# Appendix

## Appendix 1:

Online form send to the experts participating in the BaSe SCOrE survey to complete the first round of the Delphi process.


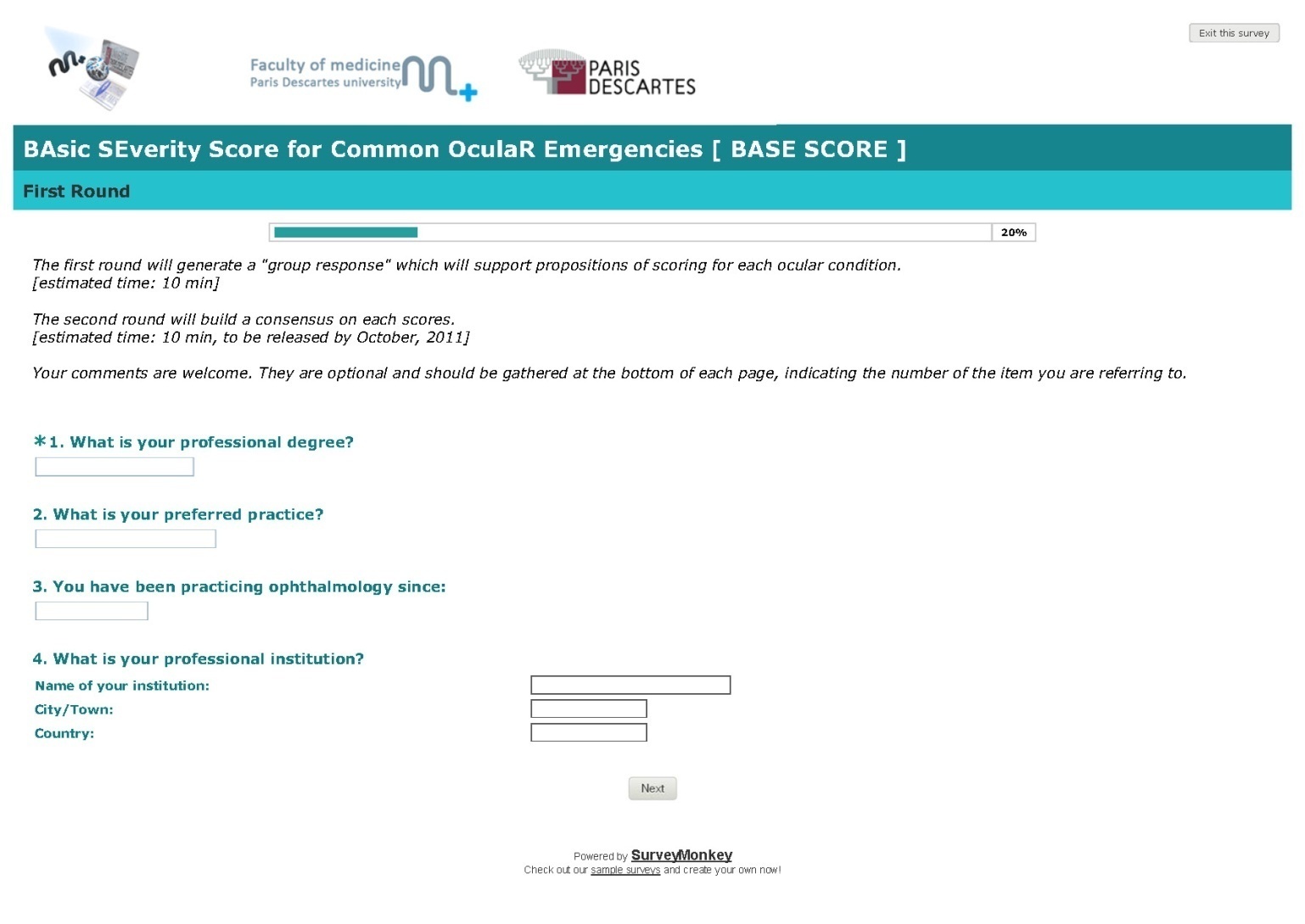

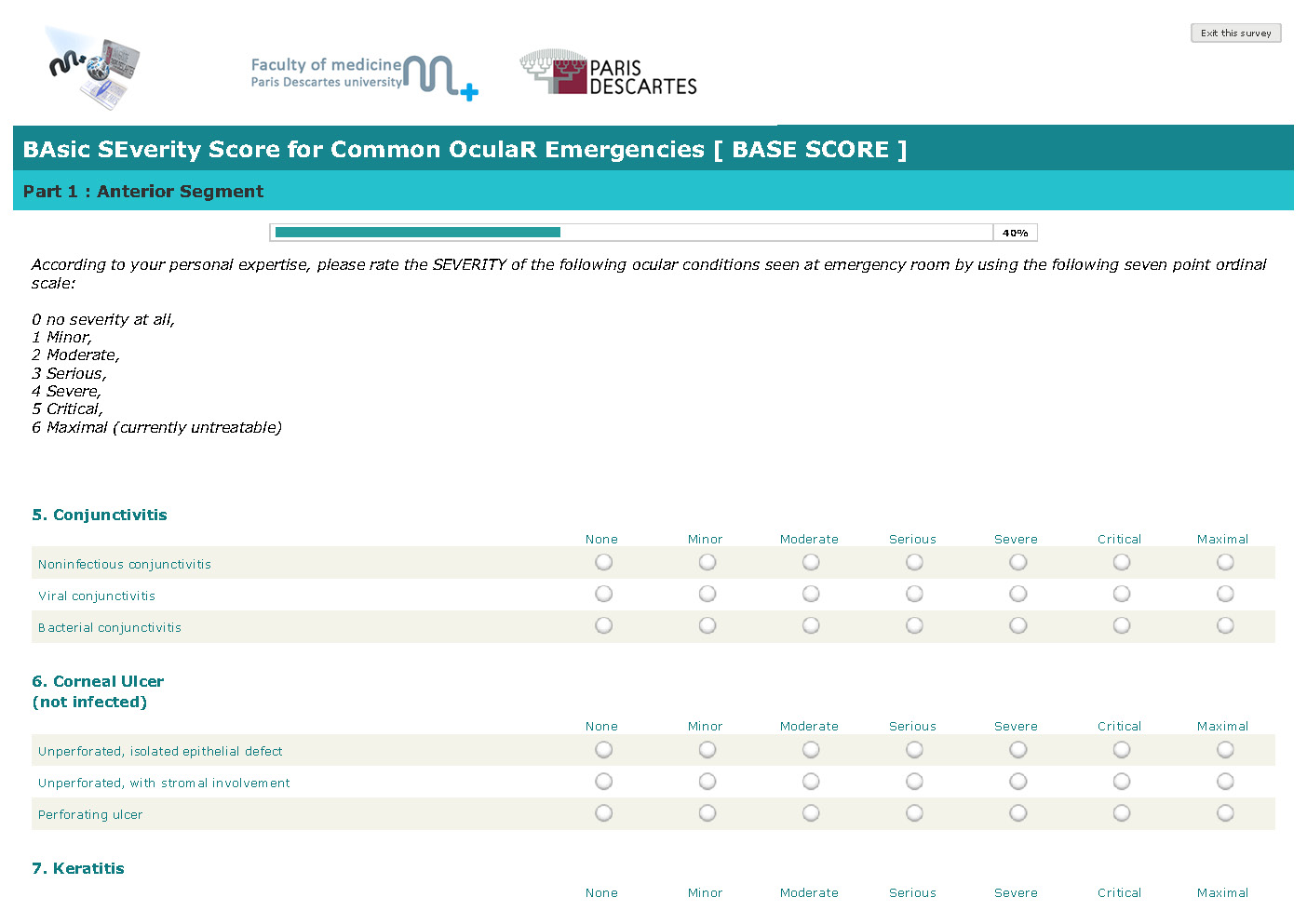

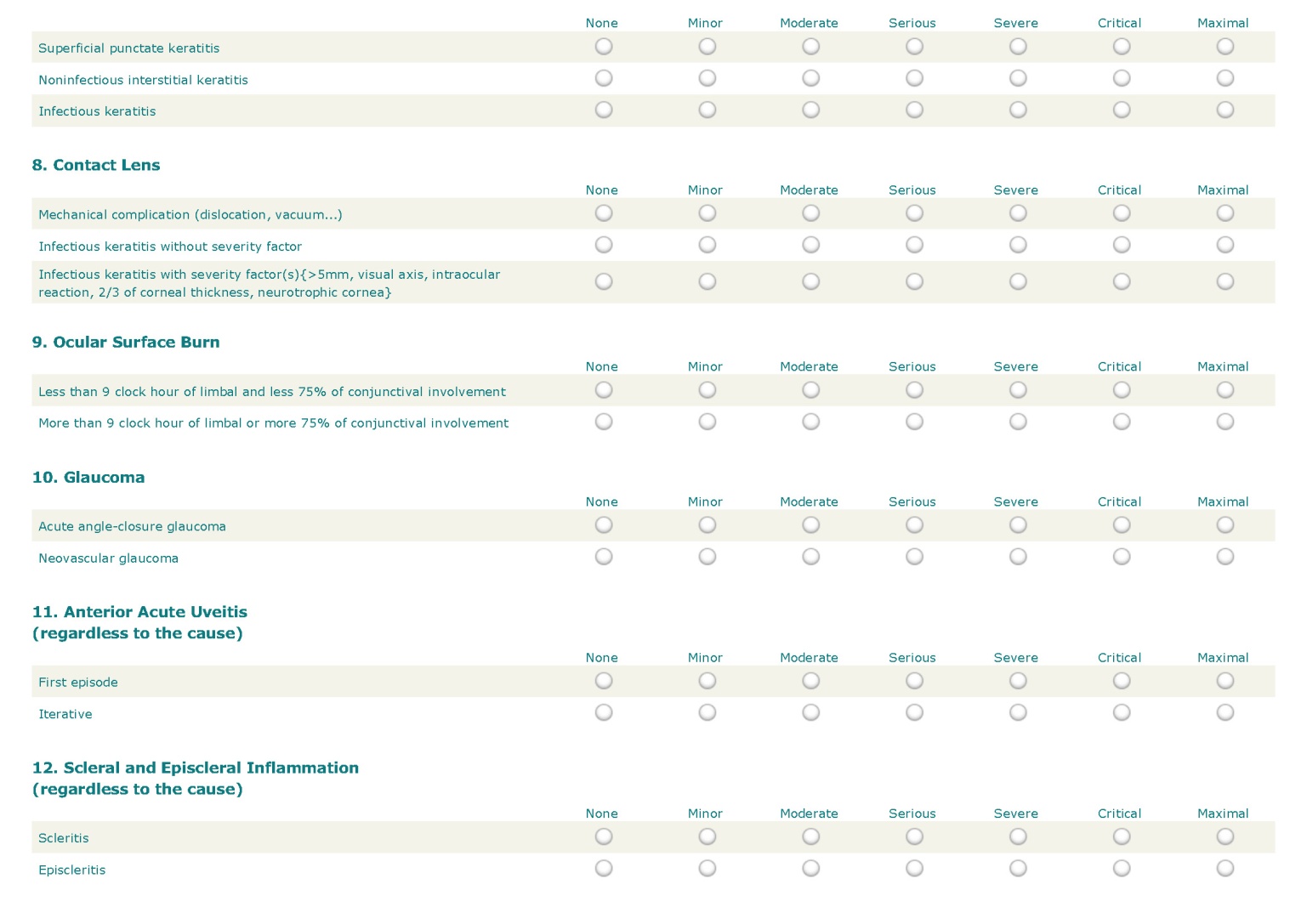

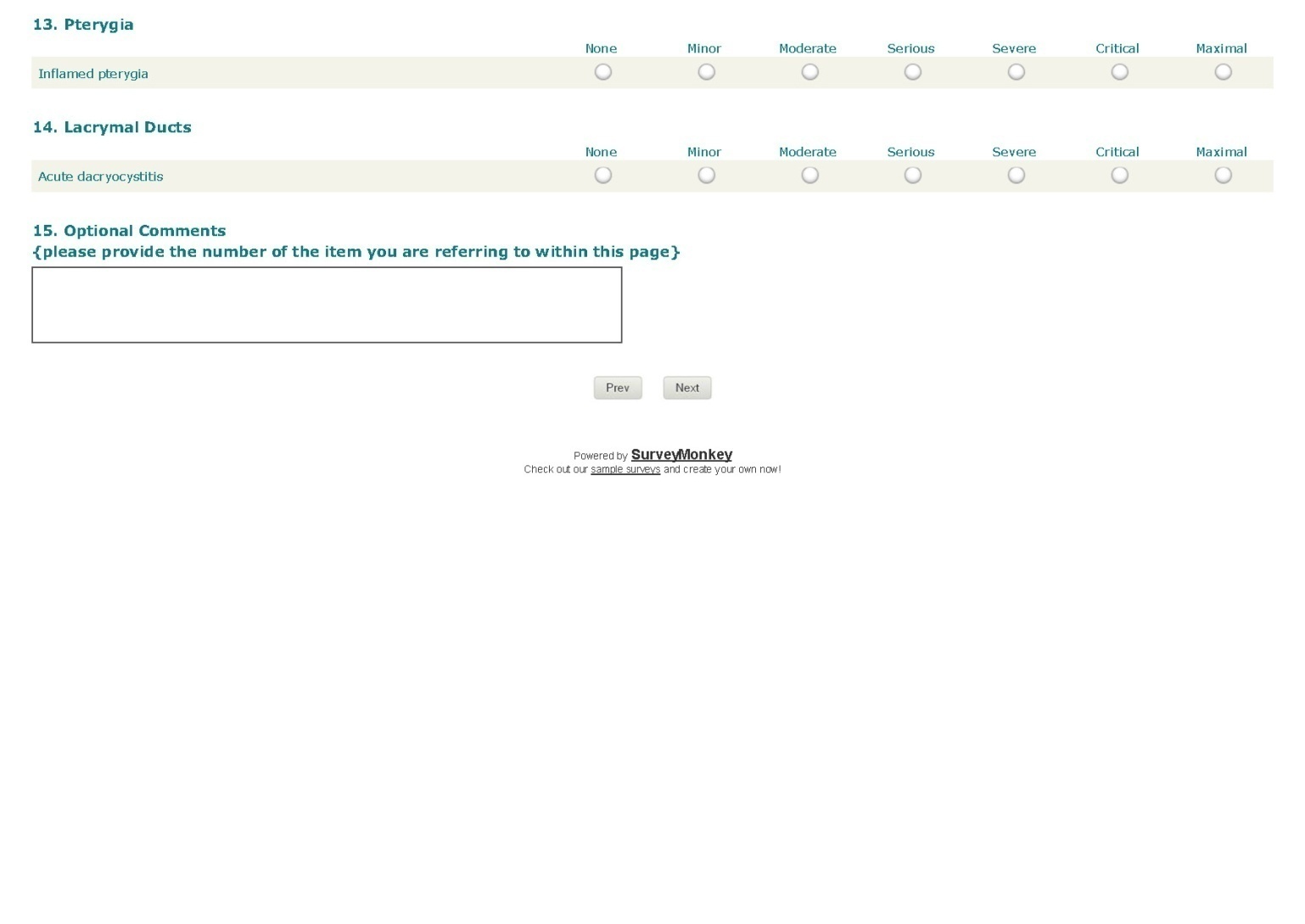

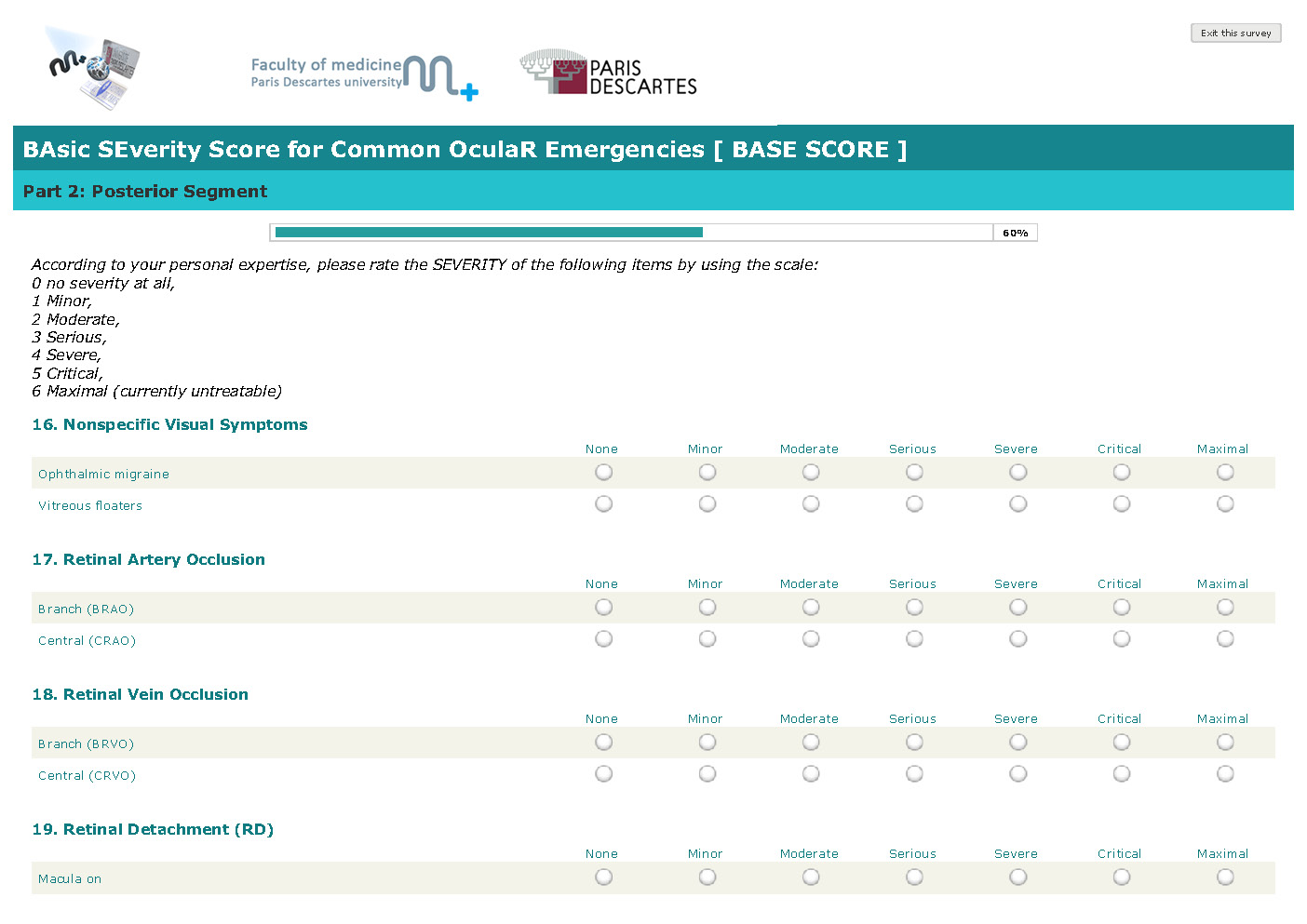

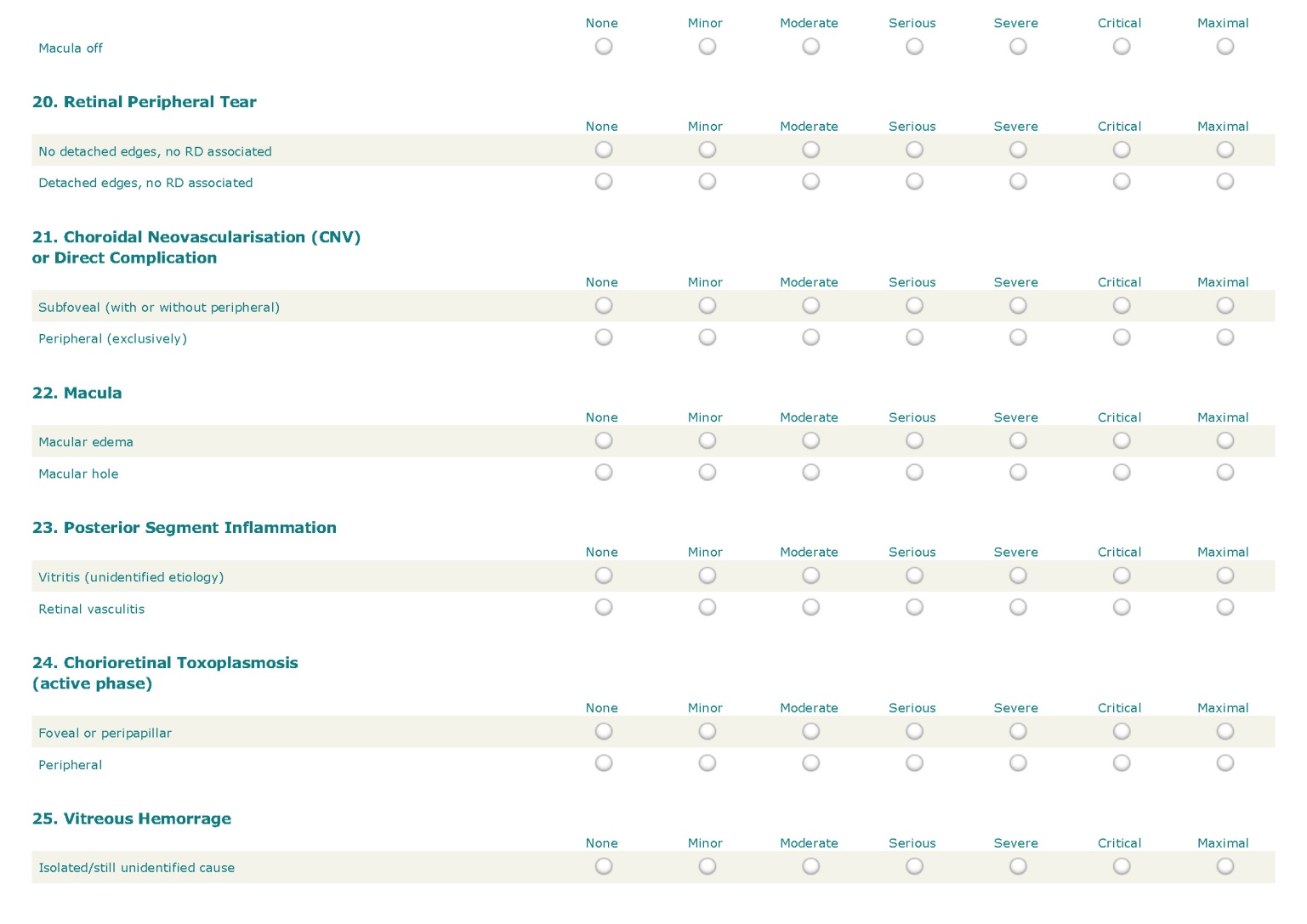

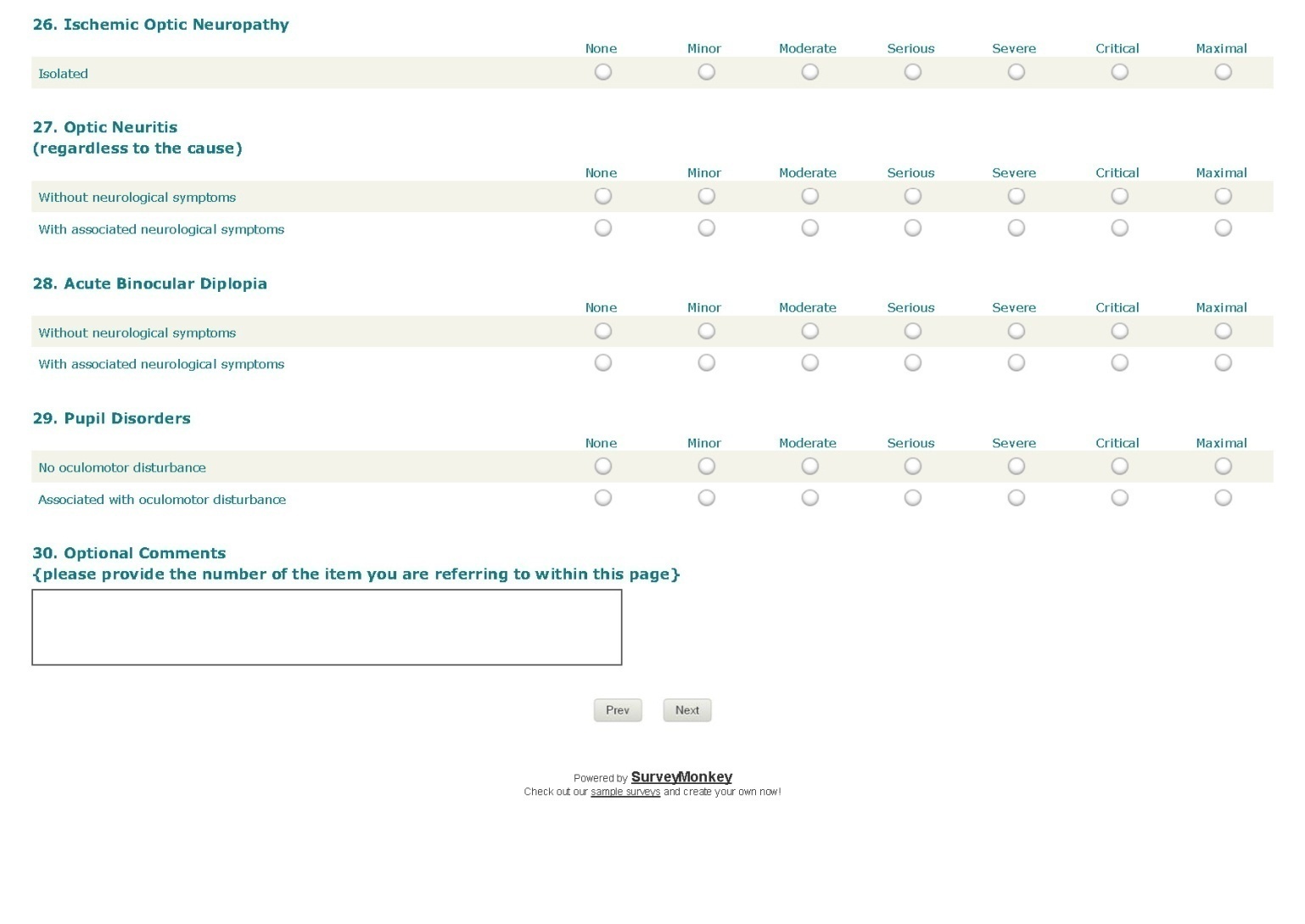

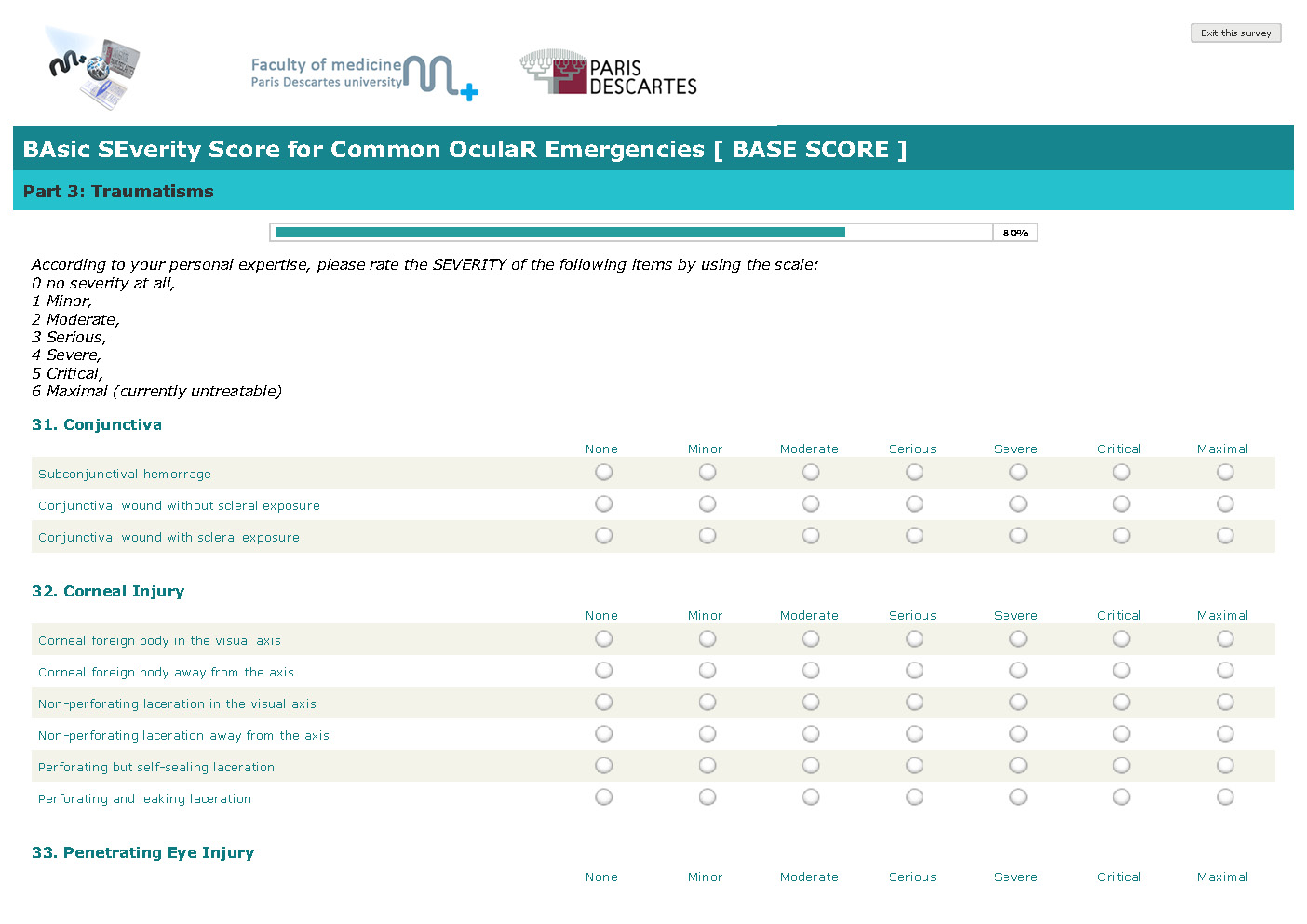

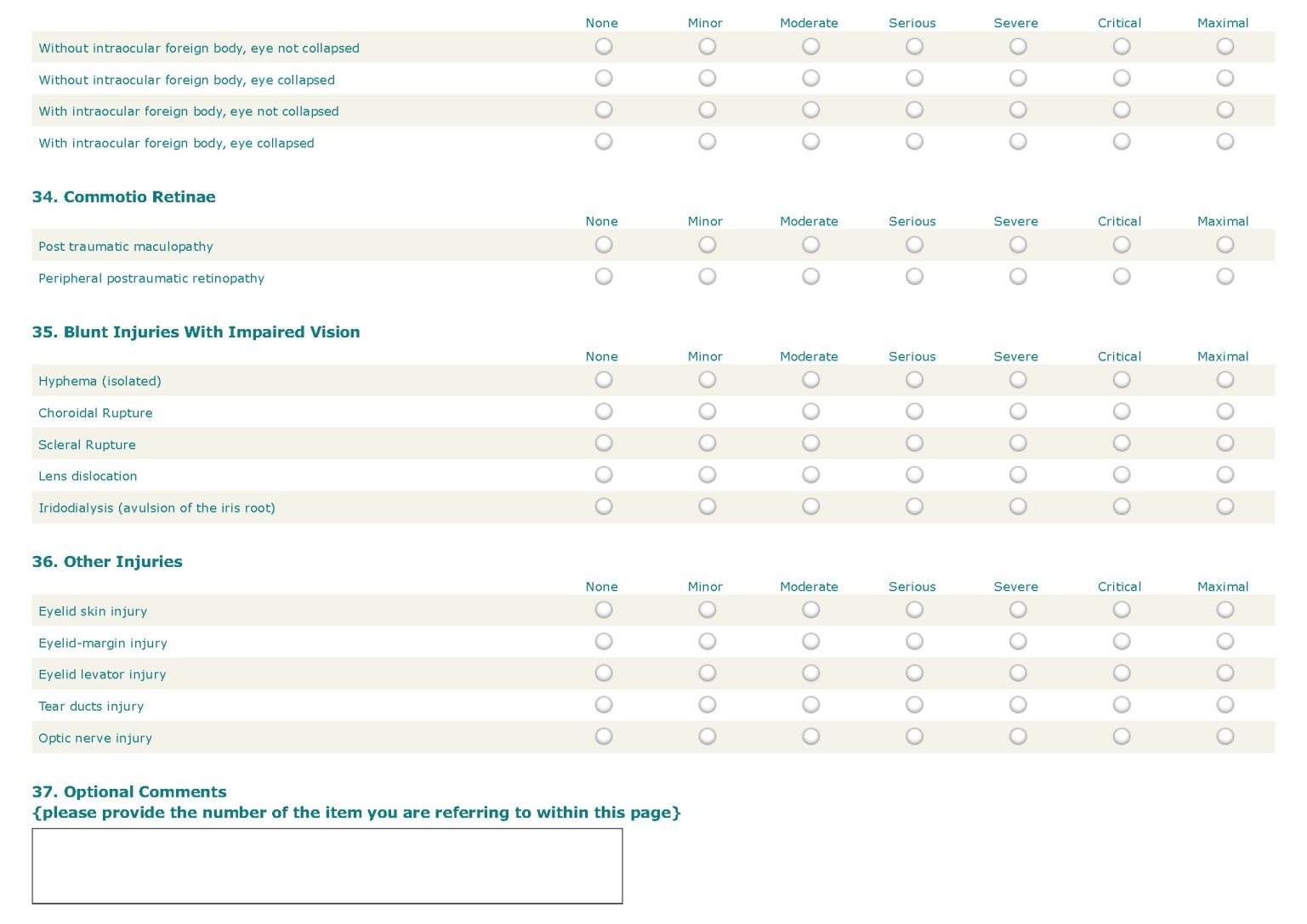

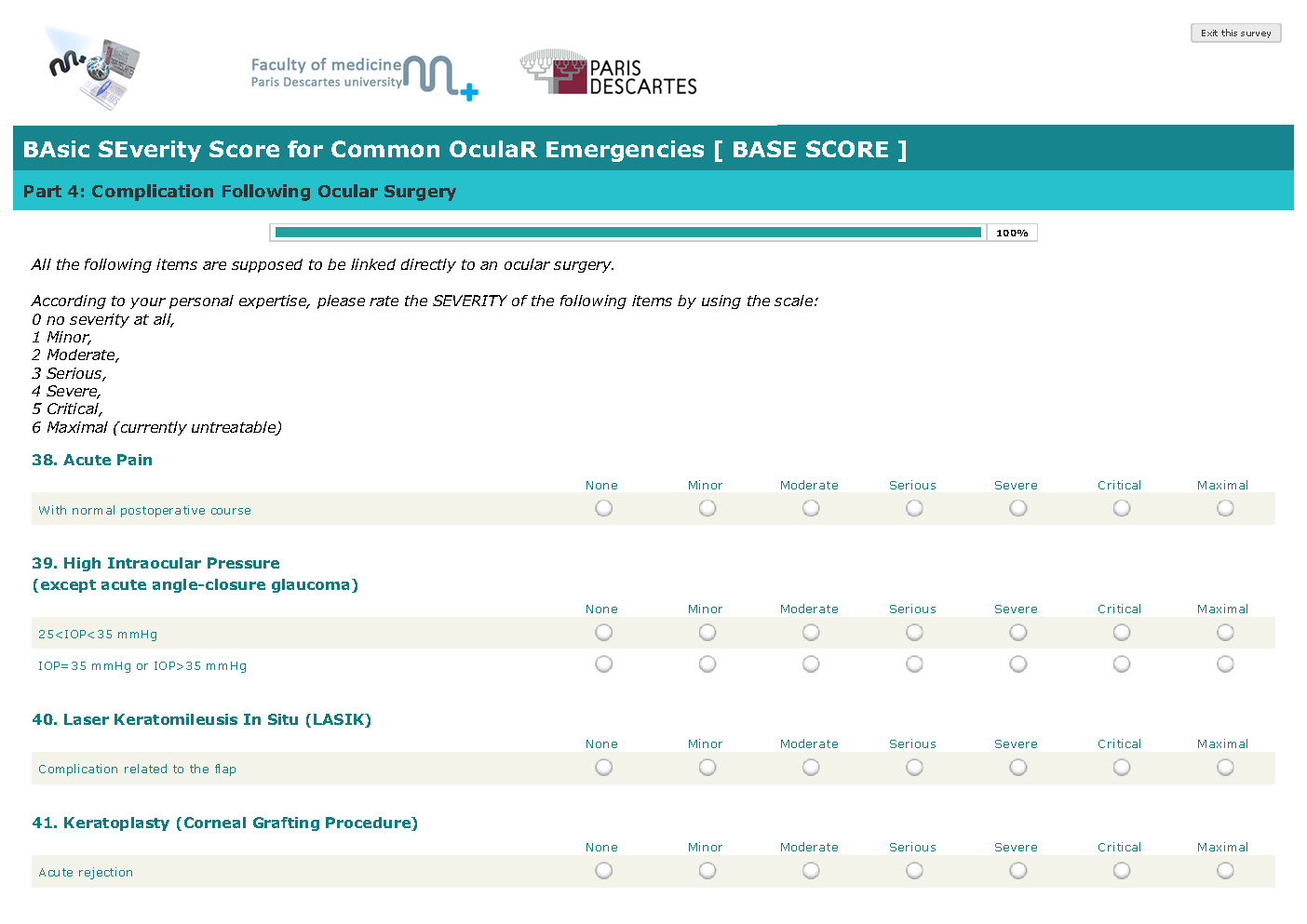

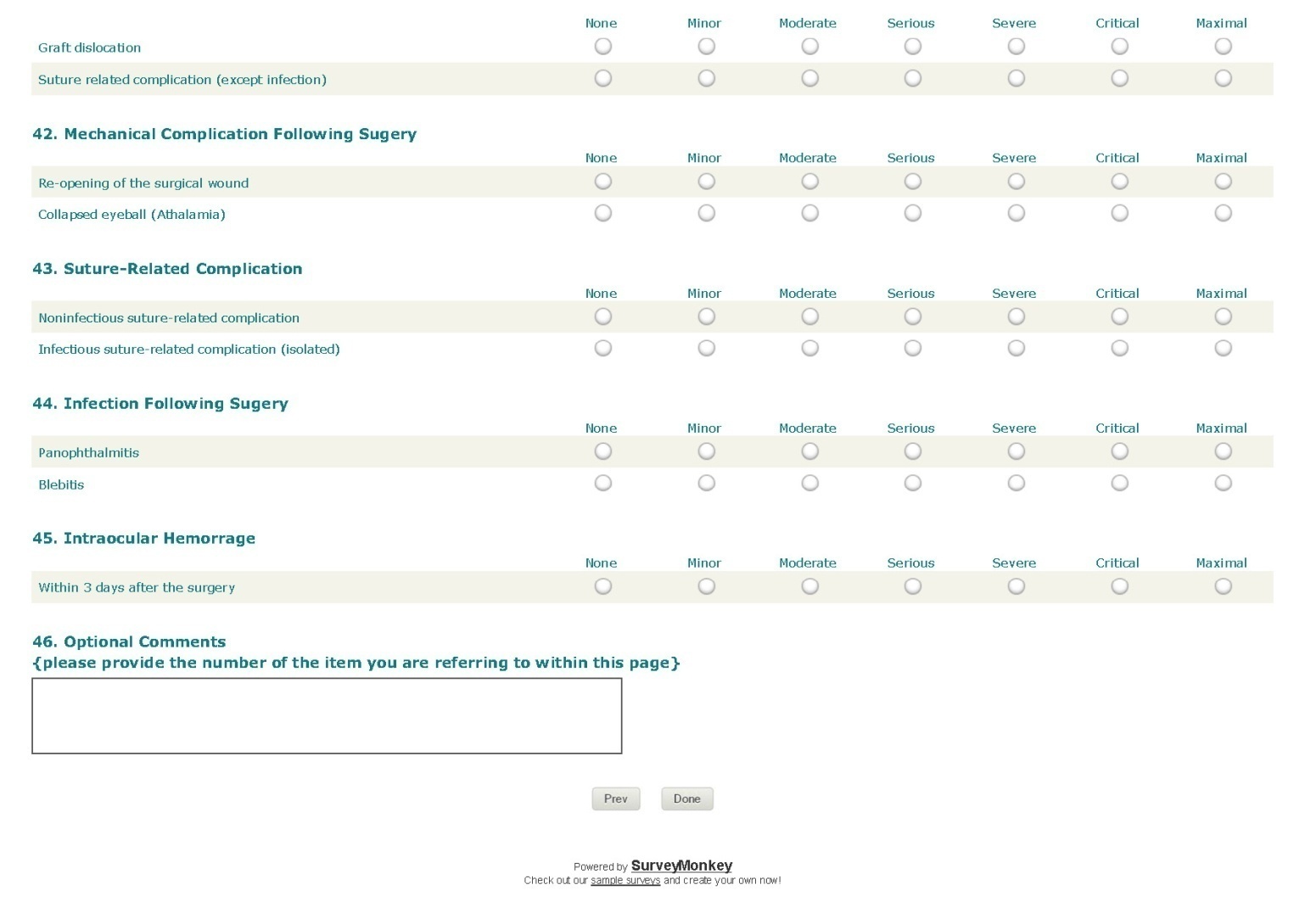


## Appendix 2:

Online form send to the experts participating in the BaSe SCOrE survey to complete the second round of the Delphi process.


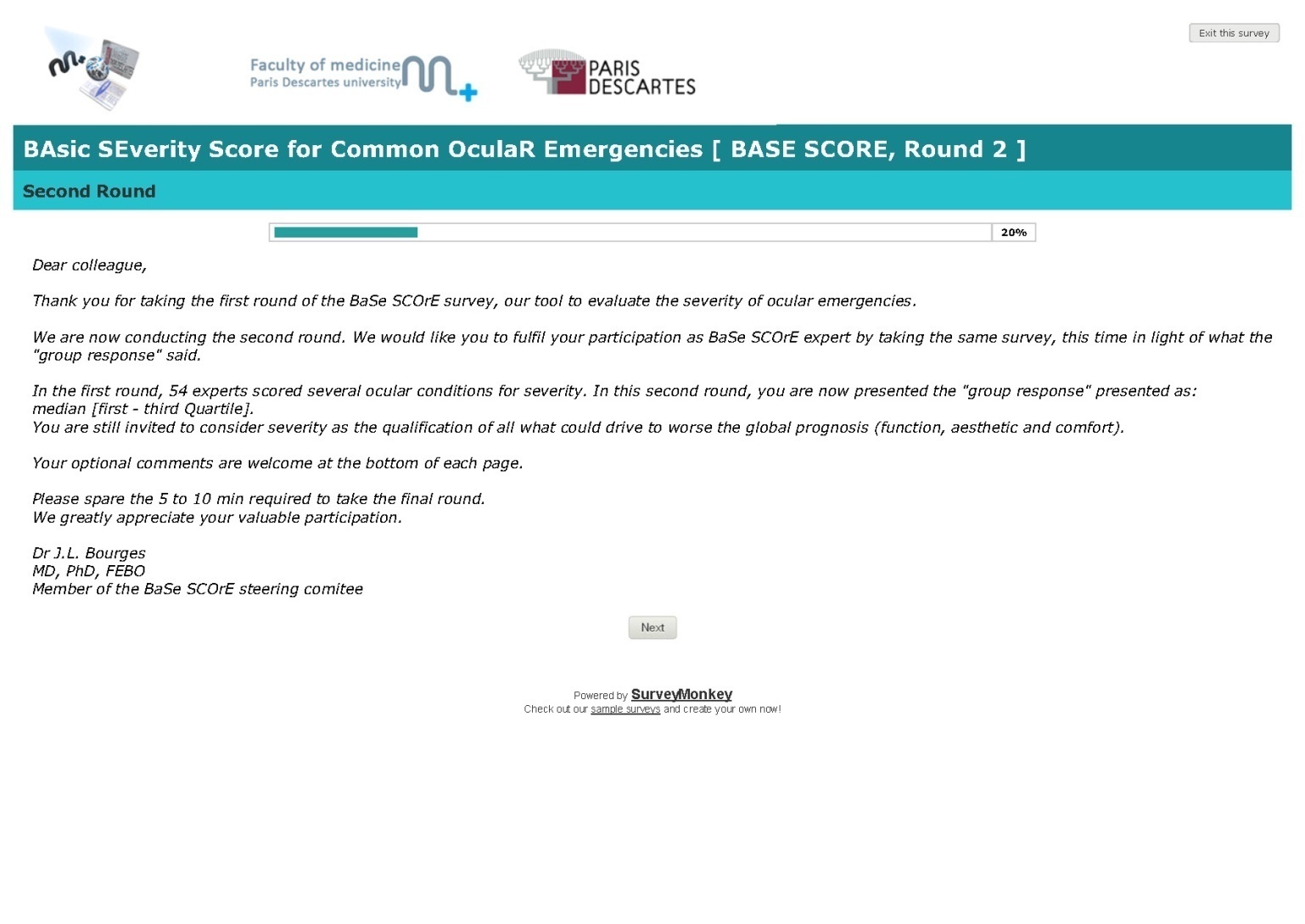

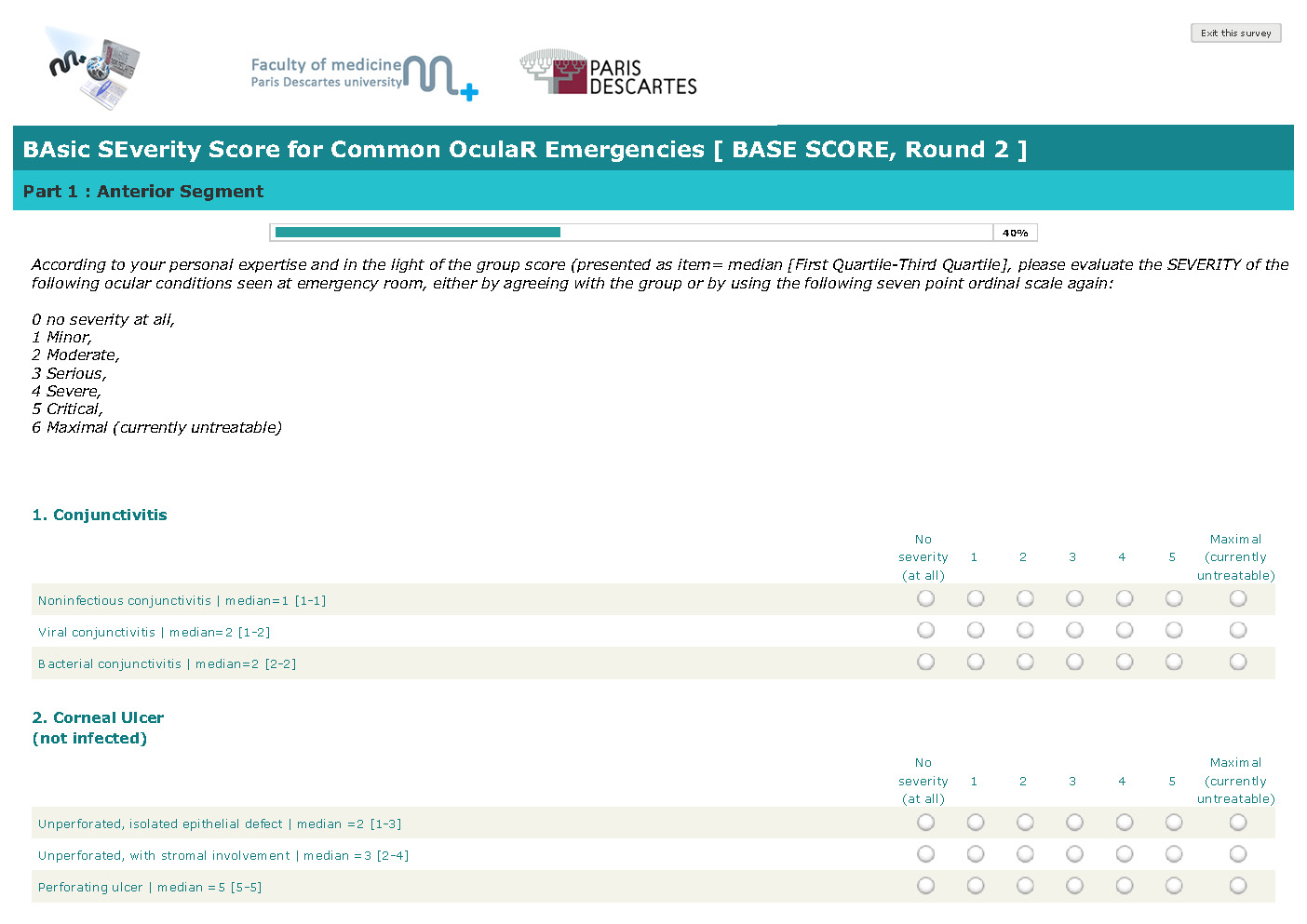

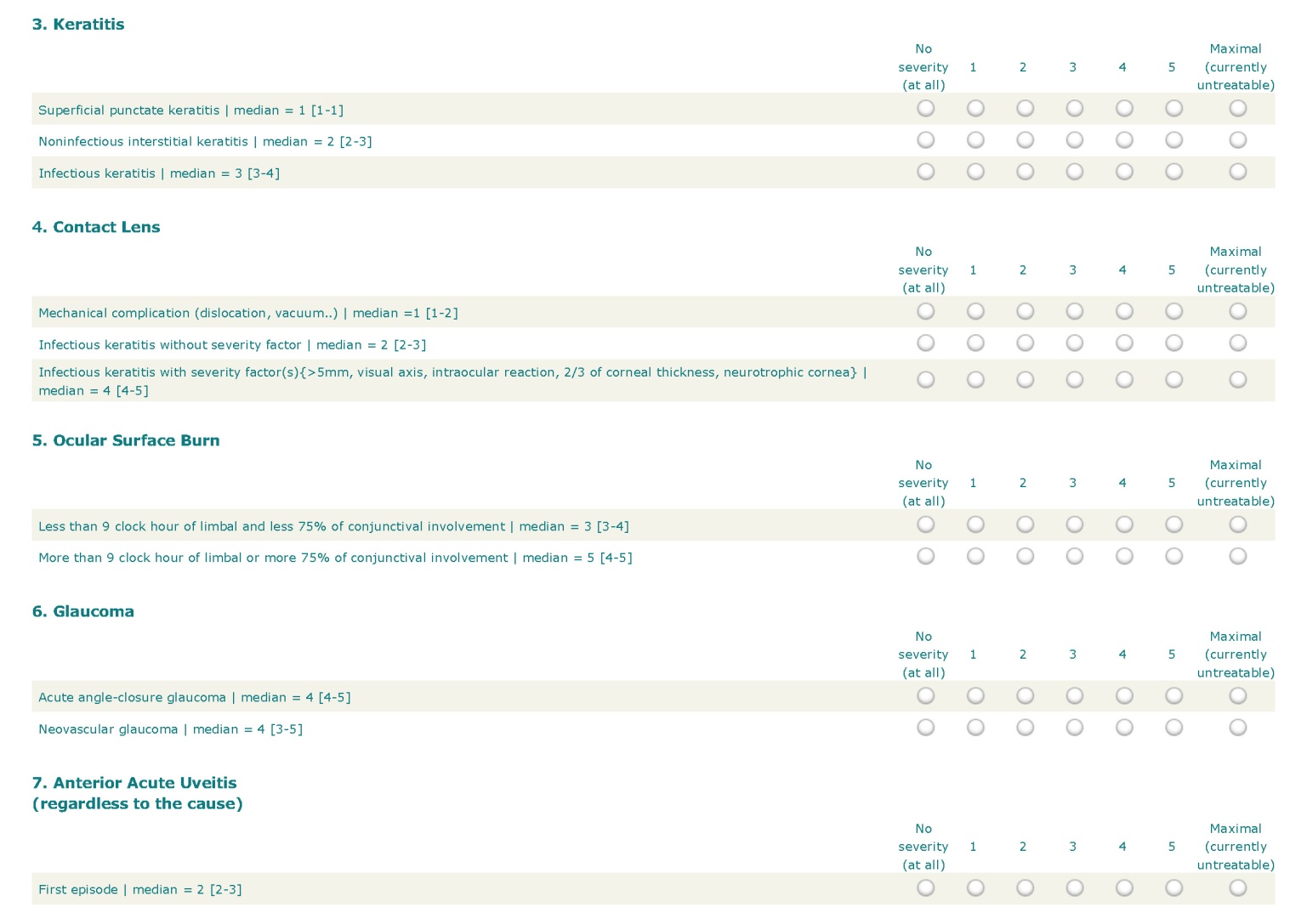

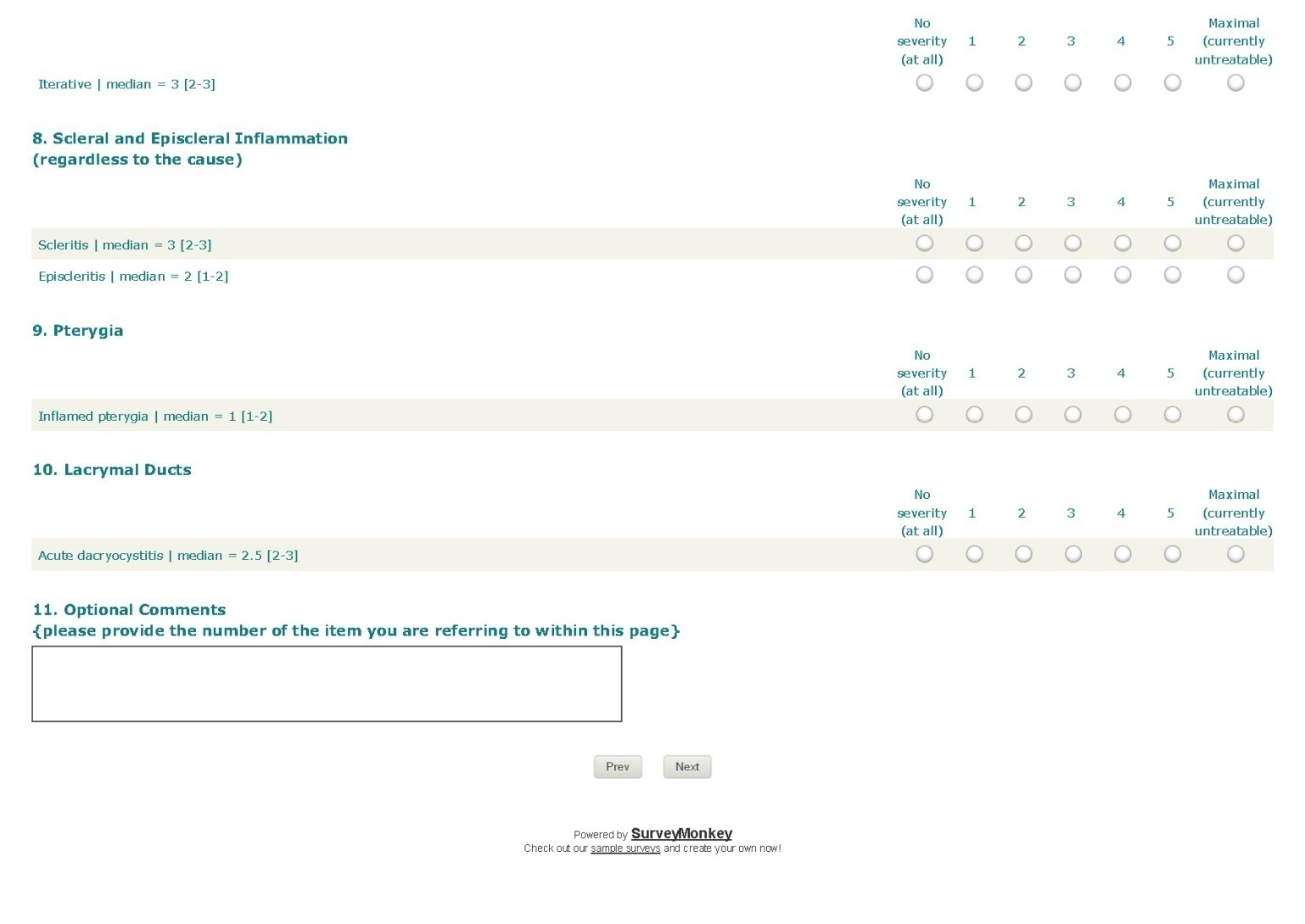

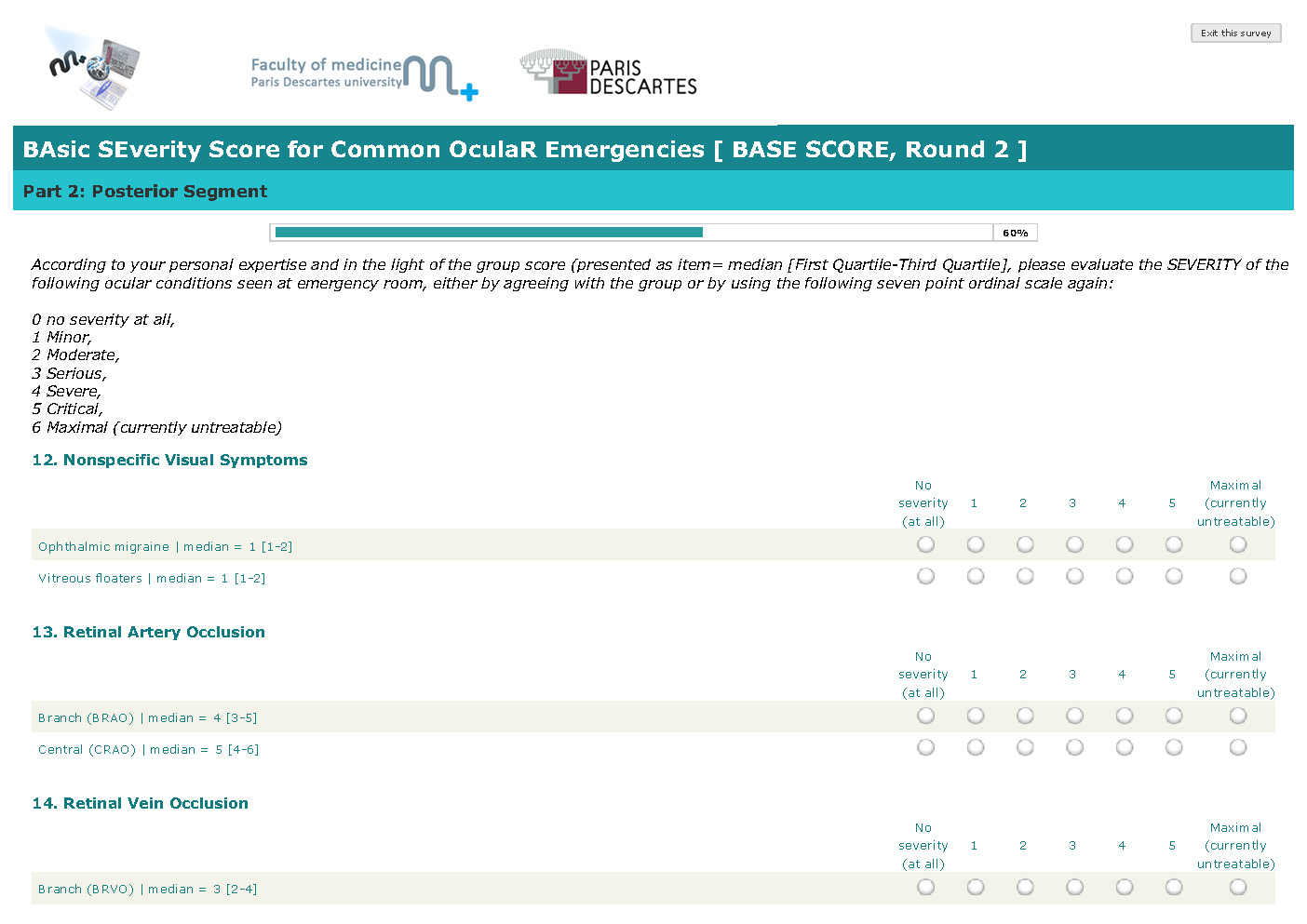

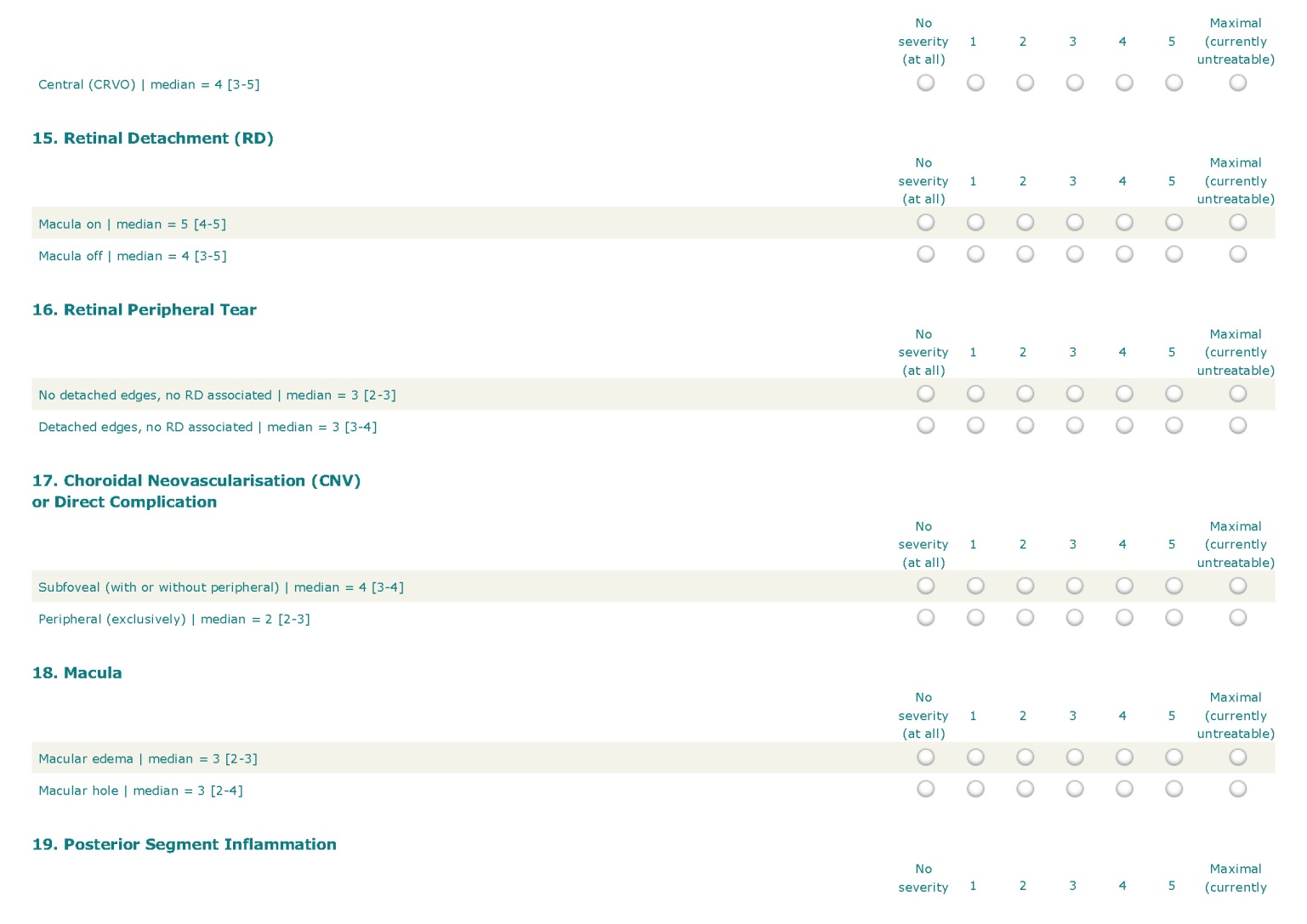

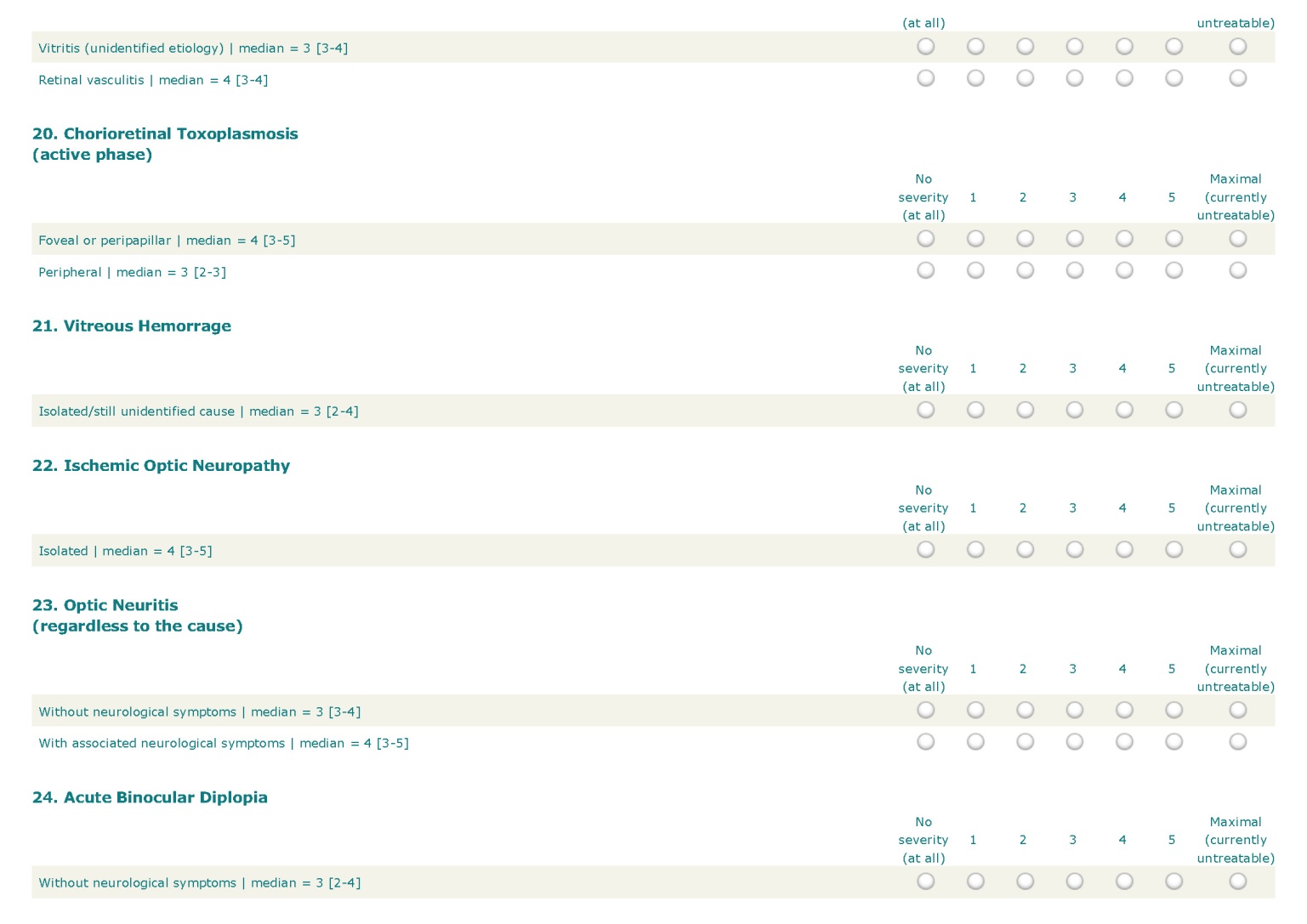

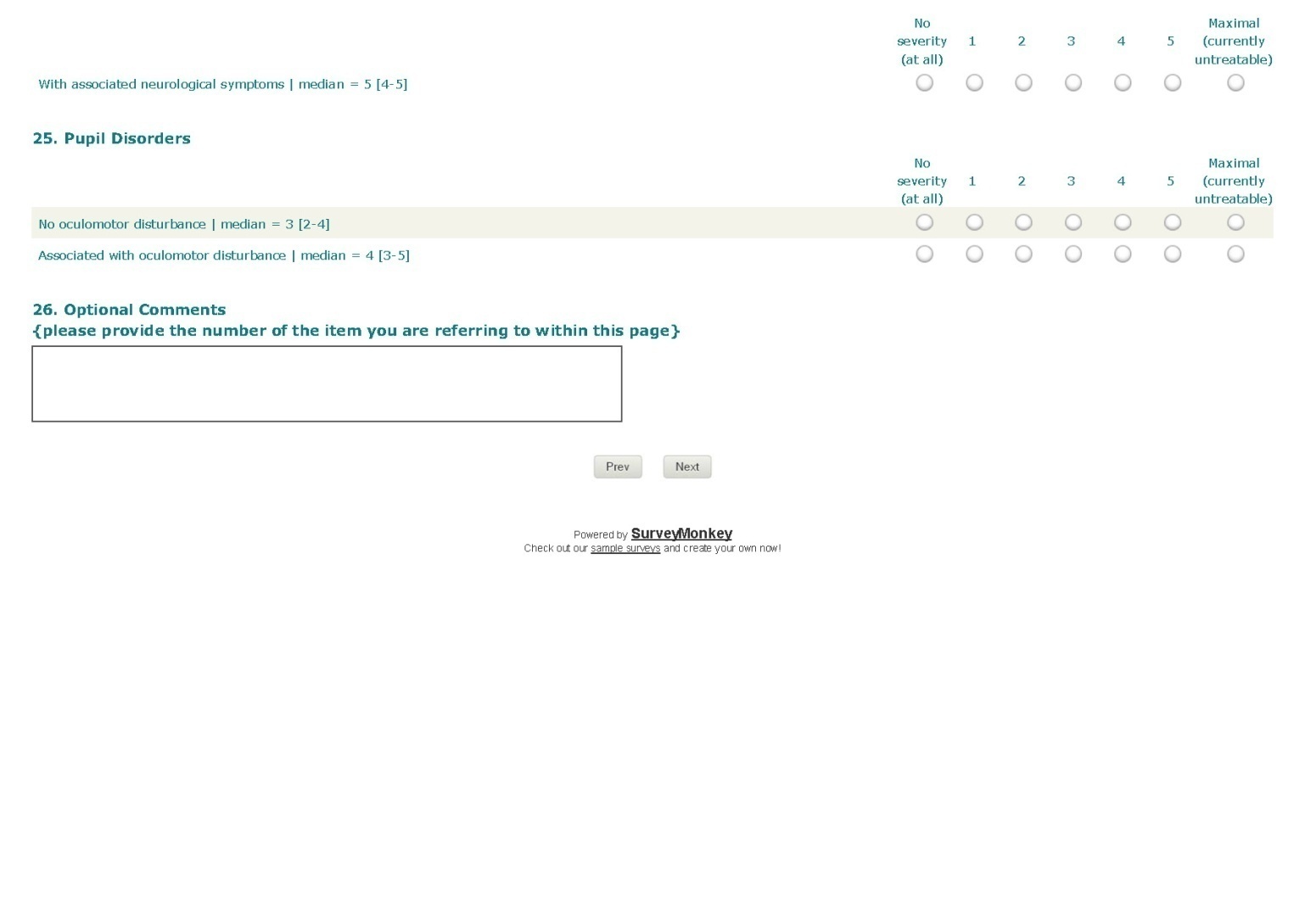

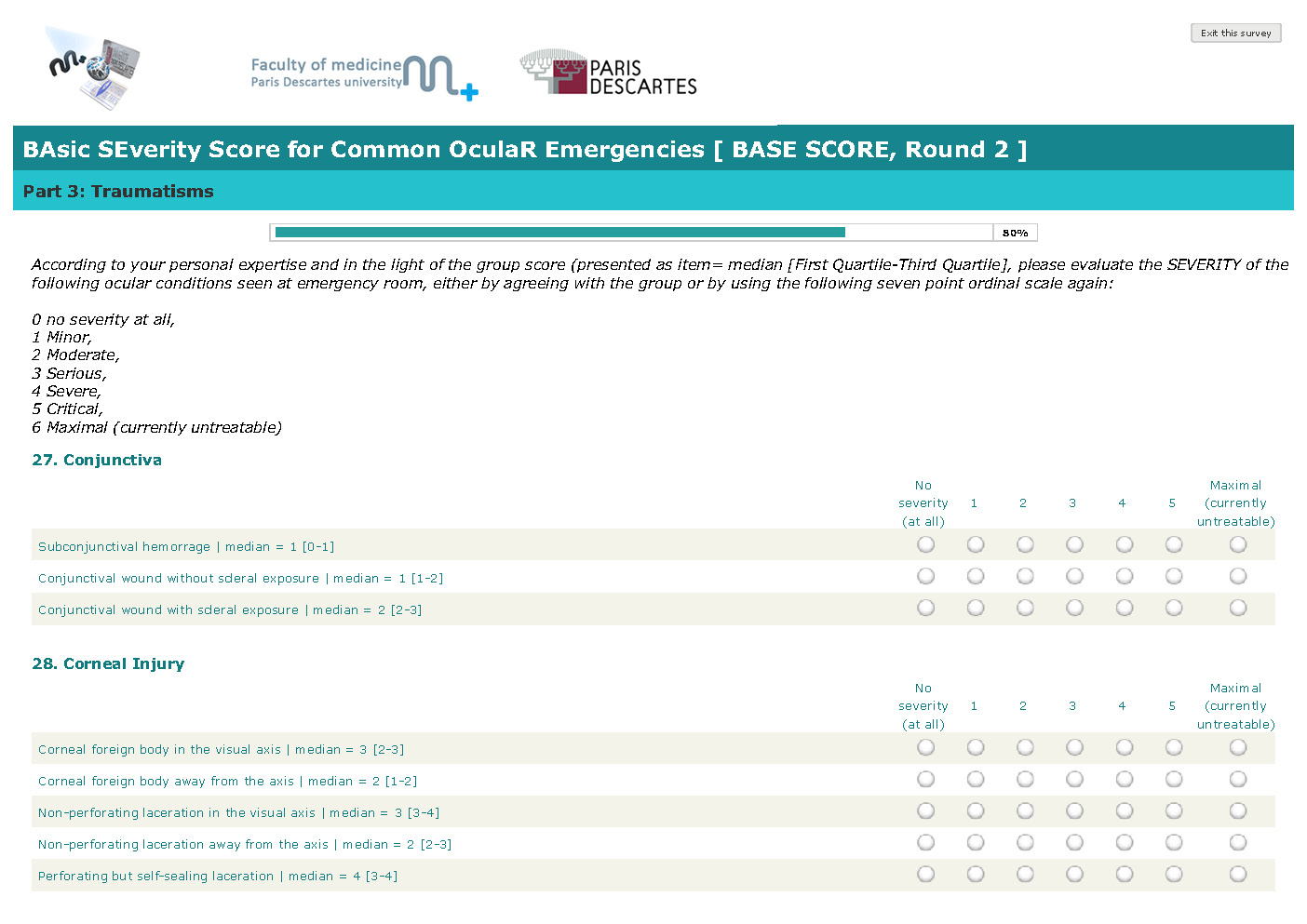

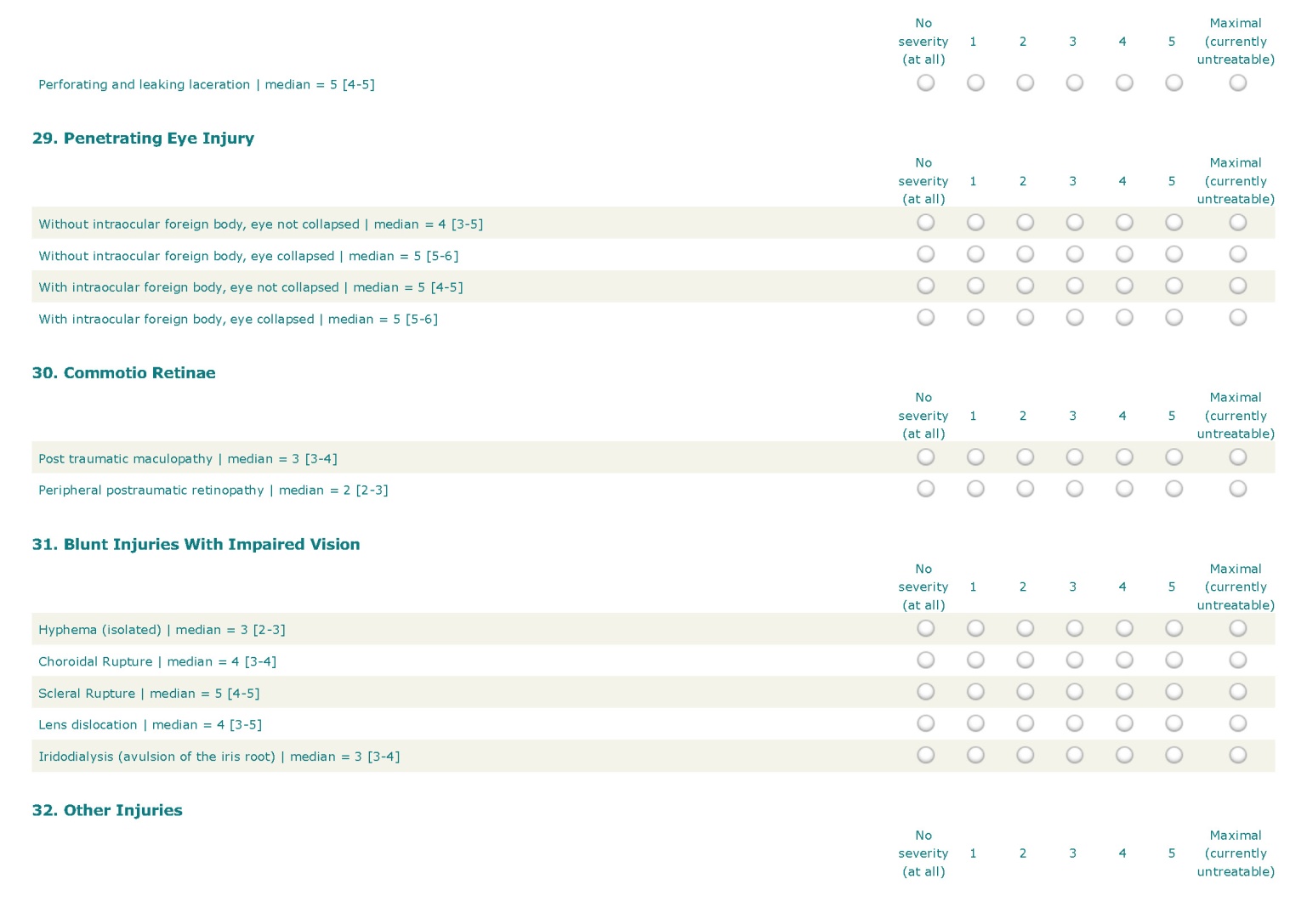

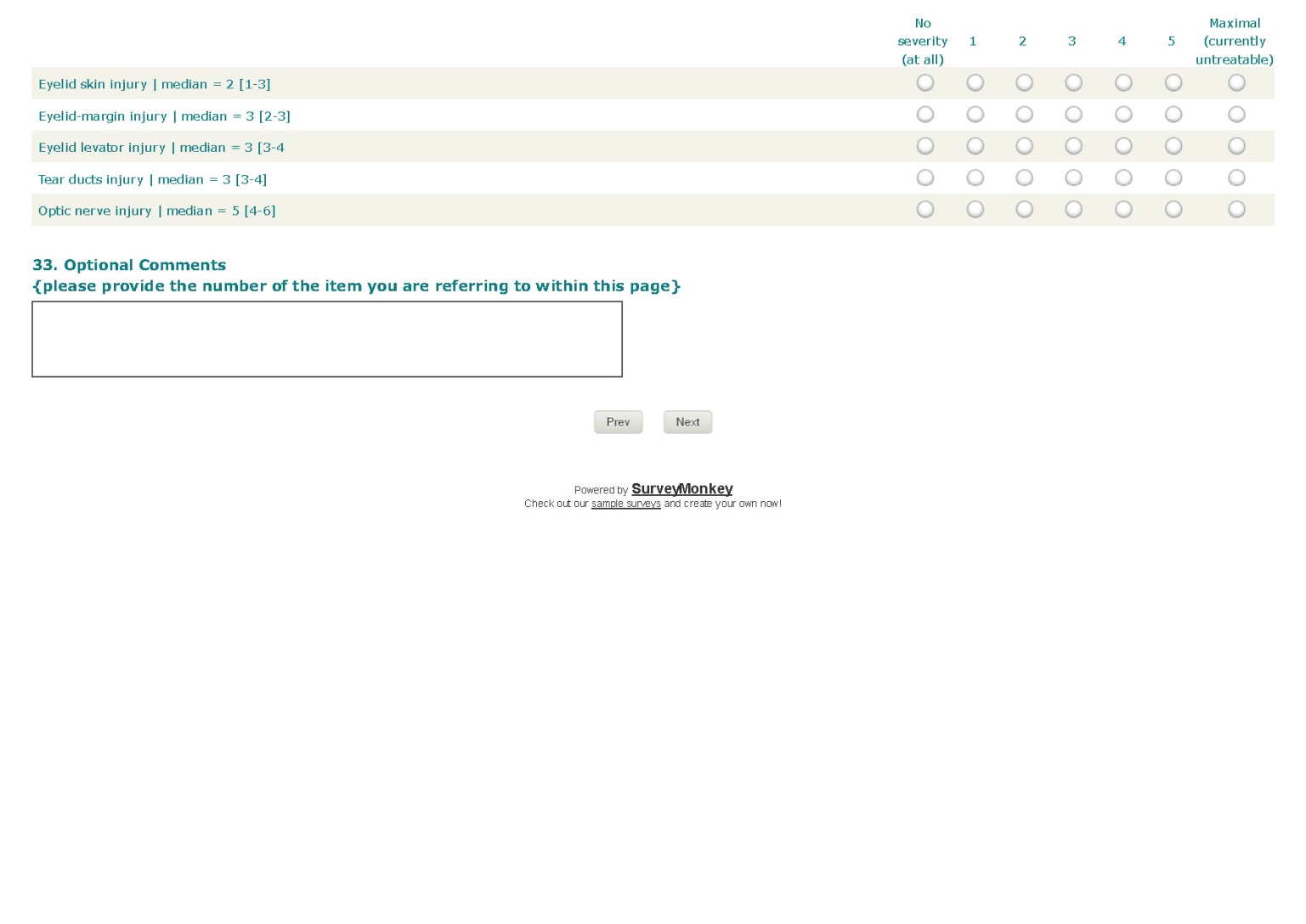

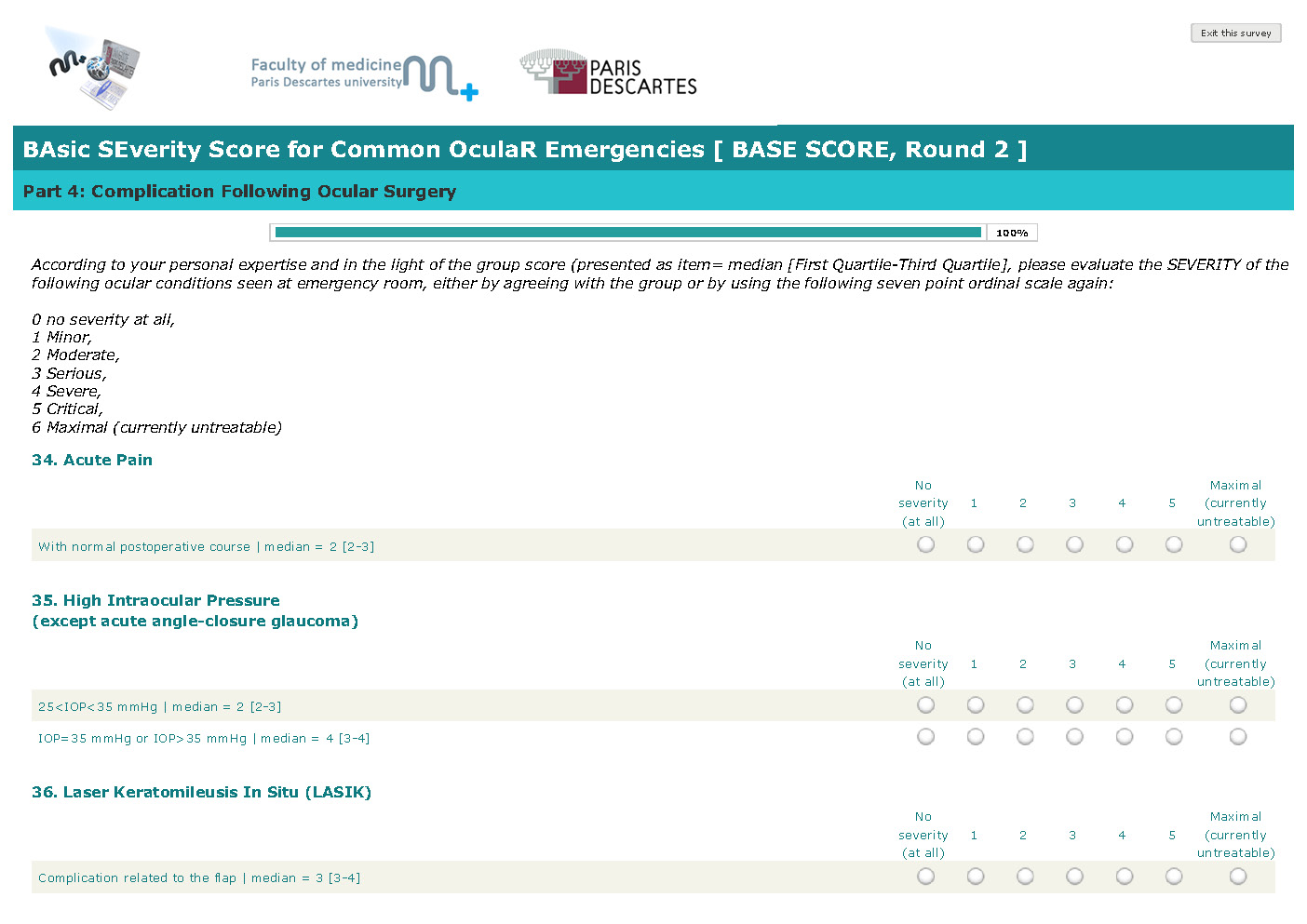

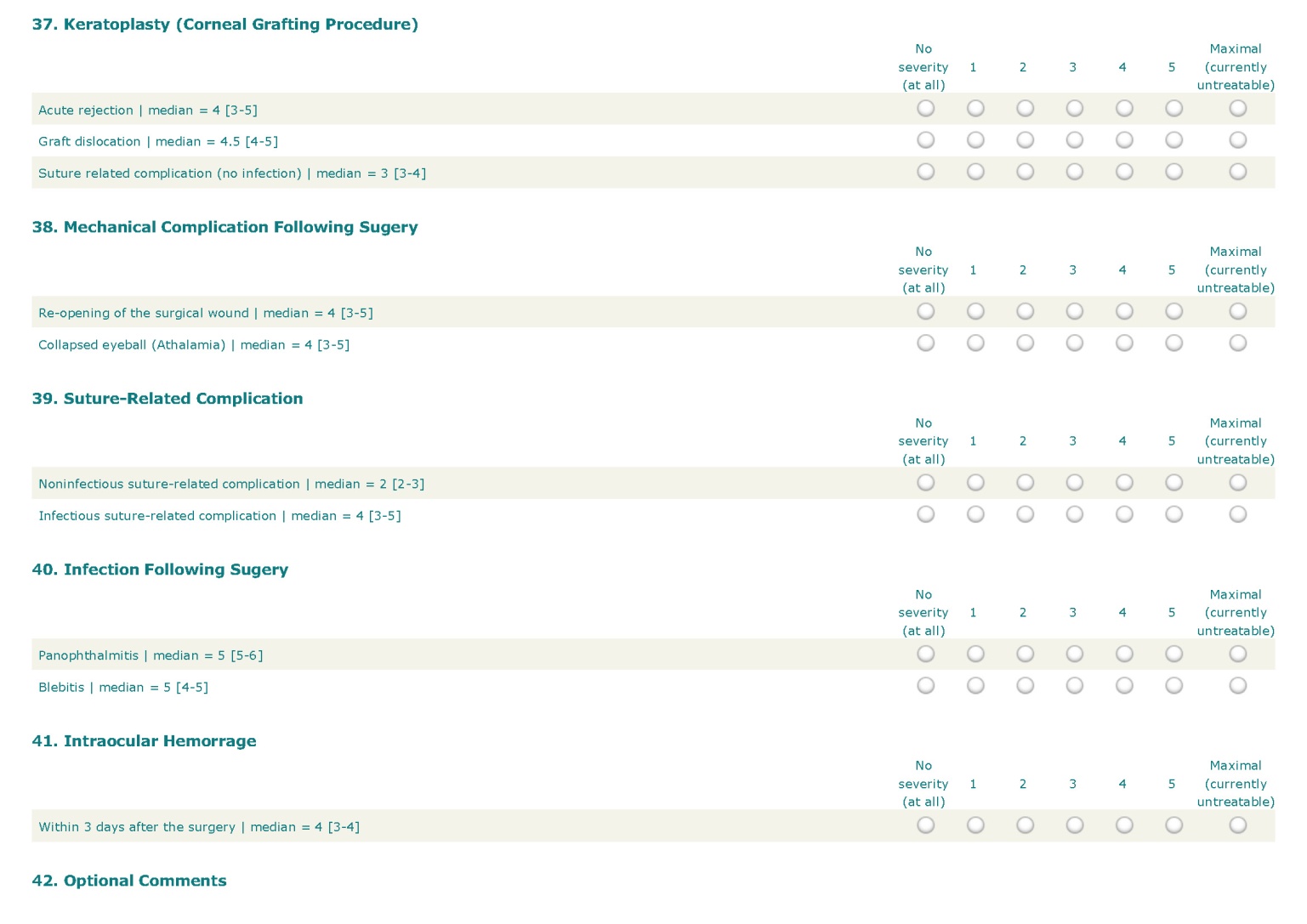

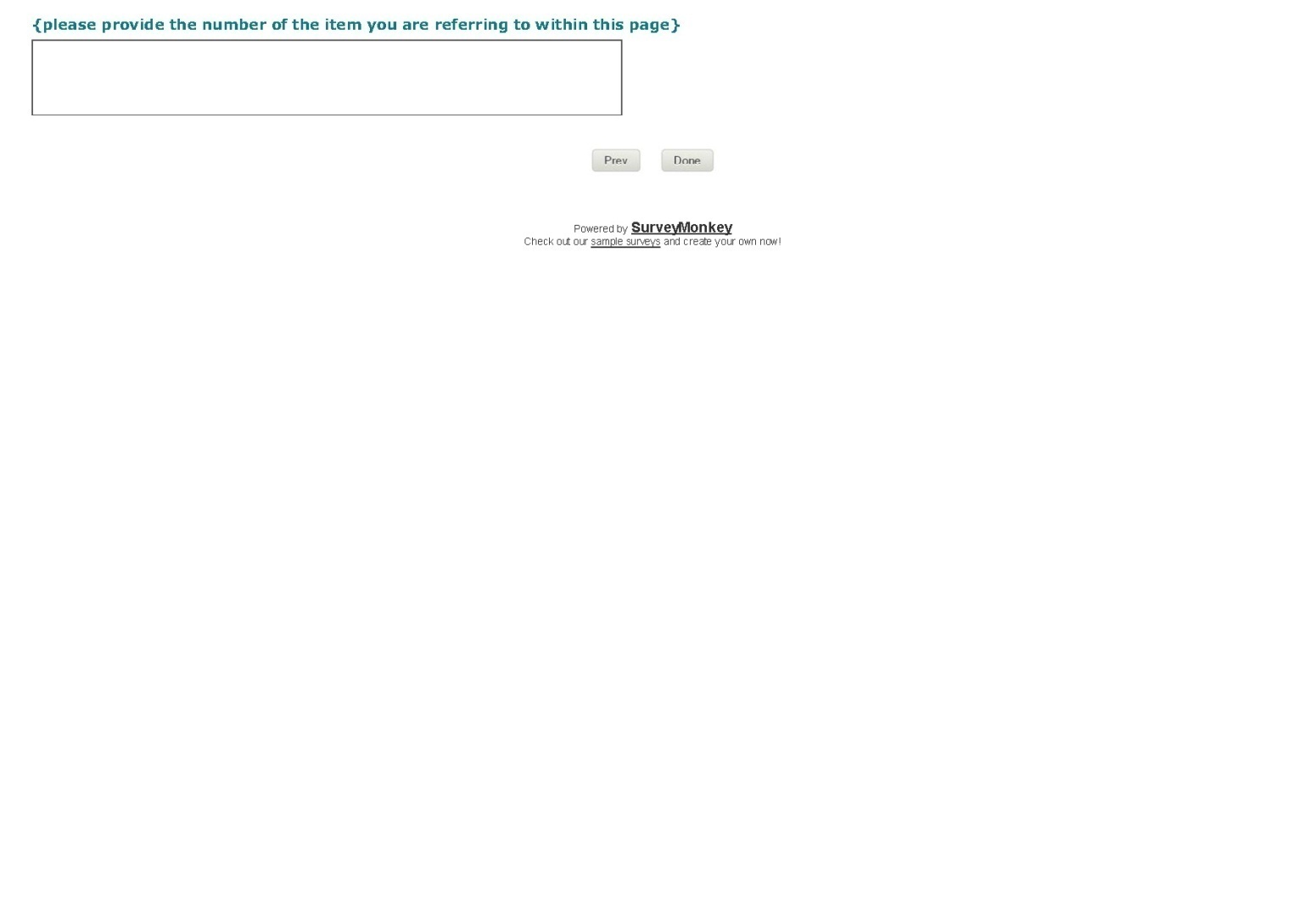

Supplement: Supplementary file 1 — The BaSe SCOrE survey consisted of an online form send to the experts. Participating experts completed the first round of the Delphi process provided in appendix 1. A second online form (appendix 2) was send to the expert based on the first form augmented with the median scores and quartiles resulting from the first round. [file 576983.f1.docx]
